# Vinculin Y822 phosphorylation regulates adhesion remodeling during cardiomyocyte maturation

**DOI:** 10.1242/jcs.263984

**Published:** 2025-12-18

**Authors:** Xiaofei Li, Rainy Wortelboer, Yi Song, Sahana Balasubramanian, Callie McLain, Alex Hernandez Manriquez, Joseph D. Suh, Brenton D. Hoffman, Adam V. Kwiatkowski, Glenn L. Radice

**Affiliations:** ^1^ Brown University Health Cardiovascular Institute, Rhode Island Hospital, Department of Medicine, Warren Alpert Medical School of Brown University, Providence, RI 02903, USA; ^2^ Department of Cell Biology, University of Pittsburgh School of Medicine, Pittsburgh, PA 15261, USA; ^3^ Department of Biomedical Engineering, Duke University, Durham, NC 27708, USA

**Keywords:** Mechanotransduction, Post-translational modification, Heart, Cardiomyocyte, Cell adhesion, Cadherin, Integrin, Adherens junctions, Focal adhesions, Fibronectin

## Abstract

In the heart, cardiomyocyte cell–matrix and cell–cell adhesions reorganize in response to increased cardiac demand and growth. Vinculin (VCL), a mechanosensitive adaptor protein, links filamentous actin to cell–matrix and cell–cell adhesions. Yet how VCL regulates remodeling of the two adhesion systems is poorly understood. Here, we investigate the role of phosphorylation at VCL tyrosine residue 822 (pY822) in cardiomyocyte adhesion and heart function. VCL Y822 phosphorylation levels peaked during adhesion remodeling in the developing heart and were reduced as adhesions matured postnatally. VCL pY822 levels also increased in the adult heart following injury. We mutated *Vcl Y822* to phenylalanine (*Y822F*) in the mouse to determine the *in vivo* function of pY822. Homozygous mutant *Vcl Y822F* mice were viable but exhibited cardiac dysfunction at 28 weeks. We found that VCL pY822 regulated cardiomyocyte cell–matrix and cell–cell adhesions during postnatal heart development. Defects in cell–cell adhesion organization were also observed in cultured *Vcl Y822F* cardiomyocytes. Our results demonstrate that VCL Y822 phosphorylation regulates adhesion organization in cardiomyocytes, highlighting the importance of post-translational modification in modulating VCL function in the heart.

## Teaser

**Summary:** Vinculin Y822 phosphorylation regulates cardiomyocyte adhesion organization and cardiac function in mice.

## INTRODUCTION

Mechanical forces play a crucial role in regulating cellular behavior and function across all cell types. The ability of a cell to sense, integrate and convert mechanical stimuli into biochemical signals that mediate intracellular changes is known as mechanotransduction. Defects in mechanotransduction are implicated in the pathogenesis of various diseases, including arteriosclerosis, cardiomyopathies, asthma and cancer (DuFort et al., 2011; Ingber, 2003; Jaalouk and Lammerding, 2009; Lyon et al., 2015). The ability to detect and respond to changes in mechanical stimuli is essential in the heart, as it enables cardiomyocytes (CMs) to react and adapt to increased mechanical load during cardiac development and disease (Lammerding et al., 2004; Majkut et al., 2014). For optimal contractility, CMs must maintain physical linkages with (1) neighboring cells, mediated by cadherin-based cell–cell adhesion complexes [i.e. adherens junctions (AJs)], and (2) the surrounding extracellular matrix (ECM), mediated by integrin-based cell–ECM adhesion complexes [i.e. focal adhesions (FAs) or costameres in muscle cells] (Israeli-Rosenberg et al., 2014; Vite and Radice, 2014; Ward and Iskratsch, 2020). Multiple cytoskeletal adaptor proteins function together to transduce mechanical stimuli across cell–cell and cell–ECM adhesion complexes (Mui et al., 2016; Zuidema et al., 2020). Among these, vinculin (VCL) is crucial in linking both AJs and FAs to the actin cytoskeleton (Bays and DeMali, 2017; Carisey and Ballestrem, 2011) and is thus well-positioned to coordinate mechanical responses in CMs*.*

VCL is a mechanosensitive protein whose primary function is to link adaptor proteins to filamentous actin (F-actin) (Atherton et al., 2016; Bays and DeMali, 2017; Leckband and de Rooij, 2014). Structurally, VCL comprises a series of helical bundles organized into five domains, termed D1–D5 (Borgon et al., 2004). D1–D4, the ‘head domain’, are connected to D5, the F-actin-binding ‘tail domain’, via a flexible linker (Choi et al., 2012; Izard et al., 2004). The head and tail domains interact to maintain VCL in a closed, auto-inhibited state, and relieving auto-inhibition is required to promote strong, load-bearing interaction with F-actin at AJs and FAs (Cohen et al., 2006; Izard et al., 2004).

Regulation of VCL activation has traditionally been thought of as binary – the auto-inhibited form is ‘opened’ by inputs (e.g. binding to one or more ligands) to create the active state. However, more recent data suggest that (1) VCL exists in multiple conformations in cells (Chorev et al., 2018) and (2) phosphorylation regulates VCL conformation (Golji et al., 2012; Huang et al., 2014; Shoyer et al., 2023). Phosphorylation sites have been mapped to all domains of VCL (Hornbeck et al., 2019). Furthermore, phosphorylation of multiple residues (e.g. Y100, Y822, Y1065, S1033 and S1054) has been shown to regulate VCL conformation at FAs and AJs (Bays and DeMali, 2017; Goldmann, 2016; Shoyer et al., 2023). Thus, VCL is better viewed as a multifaceted adaptor protein capable of sensing and responding to a combination of mechanical and biochemical cues to regulate cell adhesion.

VCL plays a crucial role in embryonic development, tissue homeostasis and disease (Sessions and Engler, 2016; Xu et al., 1998; Zemljic-Harpf et al., 2009, 2007). Mutations in the human *VCL* gene are associated with both dilated and hypertrophic forms of cardiomyopathy (Olson et al., 2002; Vasile et al., 2006a,b). In mice, global *Vcl* knockout results in mid-gestation lethality, accompanied by multiple developmental abnormalities, including cardiac defects (Xu et al., 1998). Cardiac-specific loss of *Vcl* causes sudden death (<3 months) in 50% of mutant mice, with the surviving mice eventually developing dilated cardiomyopathy and dying at 6 months of age (Zemljic-Harpf et al., 2007). At the cellular level, loss of cardiac *Vcl* causes defects in the intercalated disc (ICD) (Zemljic-Harpf et al., 2007), the specialized adhesion structure that connects adjoining CMs, and costameres (Tangney et al., 2013). However, the mechanisms by which post-translational modification regulates VCL function in cardiac physiology and disease remain unexplored.

Although VCL has numerous phosphorylation sites, the site most frequently identified by mass spectrometry is amino acid Y822 (Hornbeck et al., 2019). Furthermore, a mutation in Y822 (Y822C) has been linked to disease in humans, including uterine cancer (Cancer Genome Atlas Database; DeWane et al., 2023) and dilated cardiomyopathy (ClinVar VCV002171895.1). Here, we investigate the role of VCL Y822 phosphorylation in heart development and function in the mouse. We show that VCL pY822 levels increased with dynamic adhesion remodeling during cardiac morphogenesis, declined when AJs matured in the postnatal heart, and increased in the adult heart following injury. To define the requirement for VCL pY822 *in vivo*, we created a new strain of *Vcl* mutant mice in which Y822 was mutated to a non-phosphorylatable phenylalanine (F) using CRISPR/Cas9-mediated gene editing. Loss of pY822 disrupted the organization of CM cell adhesions in the postnatal heart, reducing AJ maturation and enhancing integrin–fibronectin expression. Defects in adhesion organization were also observed in cultured *Vcl Y822F* neonatal CMs, corroborating the *in vivo* studies. Finally, *Vcl Y822F* mice exhibited cardiac dysfunction with age. These data provide the first genetic evidence that VCL phosphorylation at Y822 regulates CM adhesion dynamics and cardiac function.

## RESULTS

### Decline in VCL pY822 levels is associated with AJ maturation in the postnatal heart

**Fig. 1.**
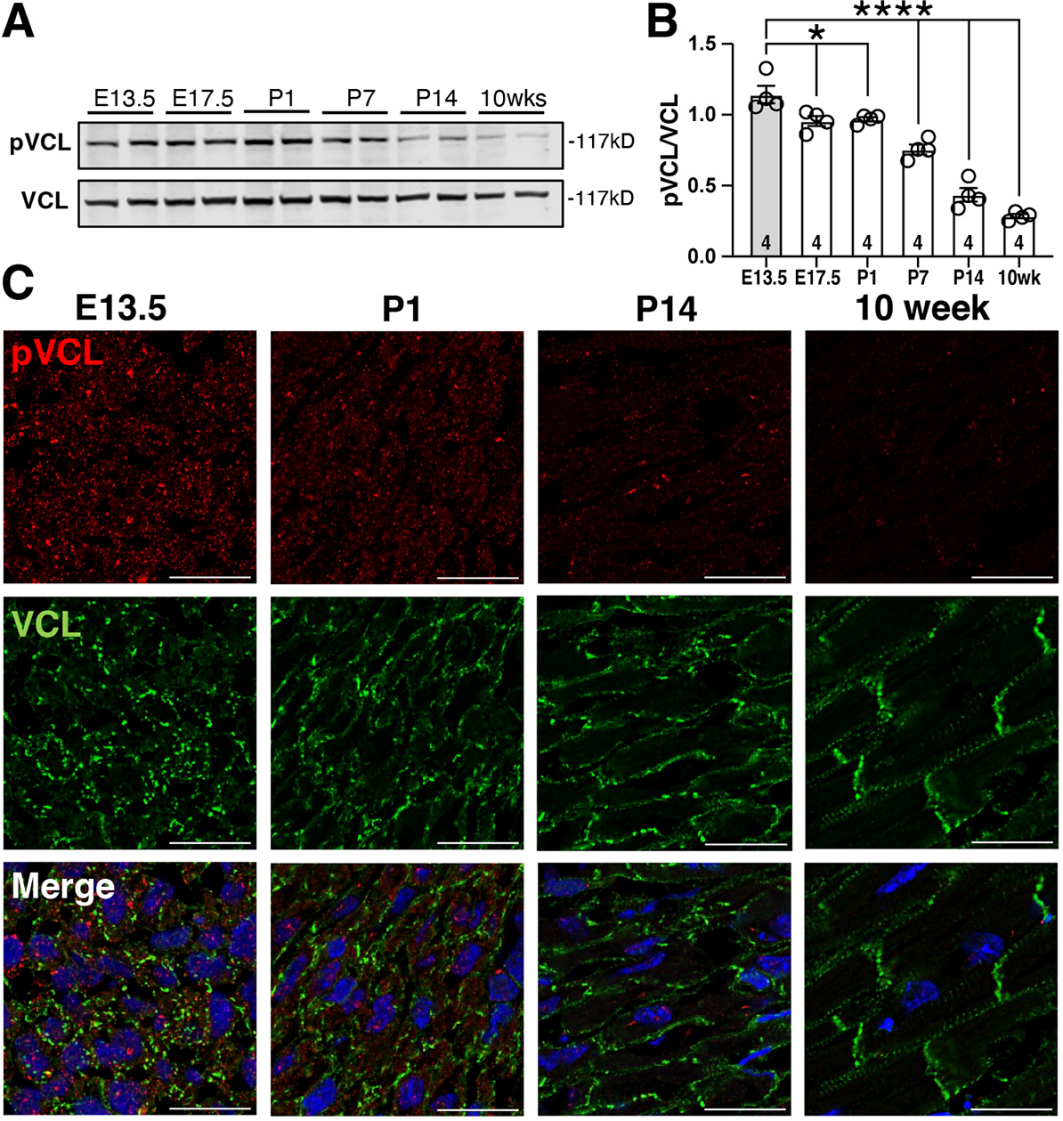
**VCL pY822 levels correlate with CM adhesion dynamics during heart development.** (A) Western blots of heart lysates from E13.5, E17.5, P1, P7, P14 and 10-week-old WT (C57BL/6J) probed for VCL pY822 and total VCL. (B) Quantification of western blots in shown A. The graph shows the ratio of Y822 phosphorylated VCL (pVCL) to total VCL (*n*=4 hearts/time point). *P<0.05, ****P<0.0001 (one-way ANOVA with Dunnett's multiple comparison to the E13.5 stage). Error bars represent s.e.m. (C) Representative WT mouse heart sections stained for pY822 VCL (red), VCL (green), and nuclei (blue). Images are deconvolved maximum projections of two-slice z-stacks (0.175 μm step size). Individual pVCL and VCL channels are shown, along with the merged image. Images representative of three independent experiments. Scale bars: 20 µm.

*In vitro* and cell-based studies have provided insights into how pY822 regulates VCL activity (Bays et al., 2014; Bertocchi et al., 2017; DeWane et al., 2023); however, the functional significance of VCL pY822 in embryonic morphogenesis and organ function remains unexplored. We focused on the heart, which experiences dynamic mechanical stresses throughout life. First, we examined the expression profile of VCL pY822 in wild-type (C57BL/6J) heart tissue using a VCL pY822-specific antibody. Western blot analysis of heart lysates revealed that VCL pY822 was abundant during the embryonic and perinatal periods but declined significantly by postnatal day (P)14 and remained low in 10-week-old adult mice (Fig. 1A,B). Next, we investigated the localization of VCL pY822 via immunostaining. Heart sections from embryonic day (E)13.5, P1, P14 and 10-week-old adult mice were stained for VCL pY822 and VCL (Fig. 1C). VCL marked the nascent adhesions of developing CMs (E13.5 and P1), the organizing terminal and lateral membranes of maturing CMs (P14), and the ICDs and costameres of mature CMs (10 weeks). VCL pY822, by contrast, was predominantly punctate and cytoplasmic (Fig. 1C). VCL pY822 staining intensity was highest at E13.5 and decreased with age, matching the western blots results (Fig. 1A,B). During heart development and the early stages of CM maturation (E13.5–P1), dynamic adhesions play a crucial role in CM rearrangement and integration during chamber expansion (Miao et al., 2019, 2024). In the heart, VCL pY822 levels are highest during these stages, but steadily decrease as adhesive structures mature in the postnatal heart. Given this developmental timing, we speculate that Y822 phosphorylation regulates VCL function at dynamic adhesions.

### Increased VCL Y822 phosphorylation in the adult heart following myocardial injury

**Fig. 2.**
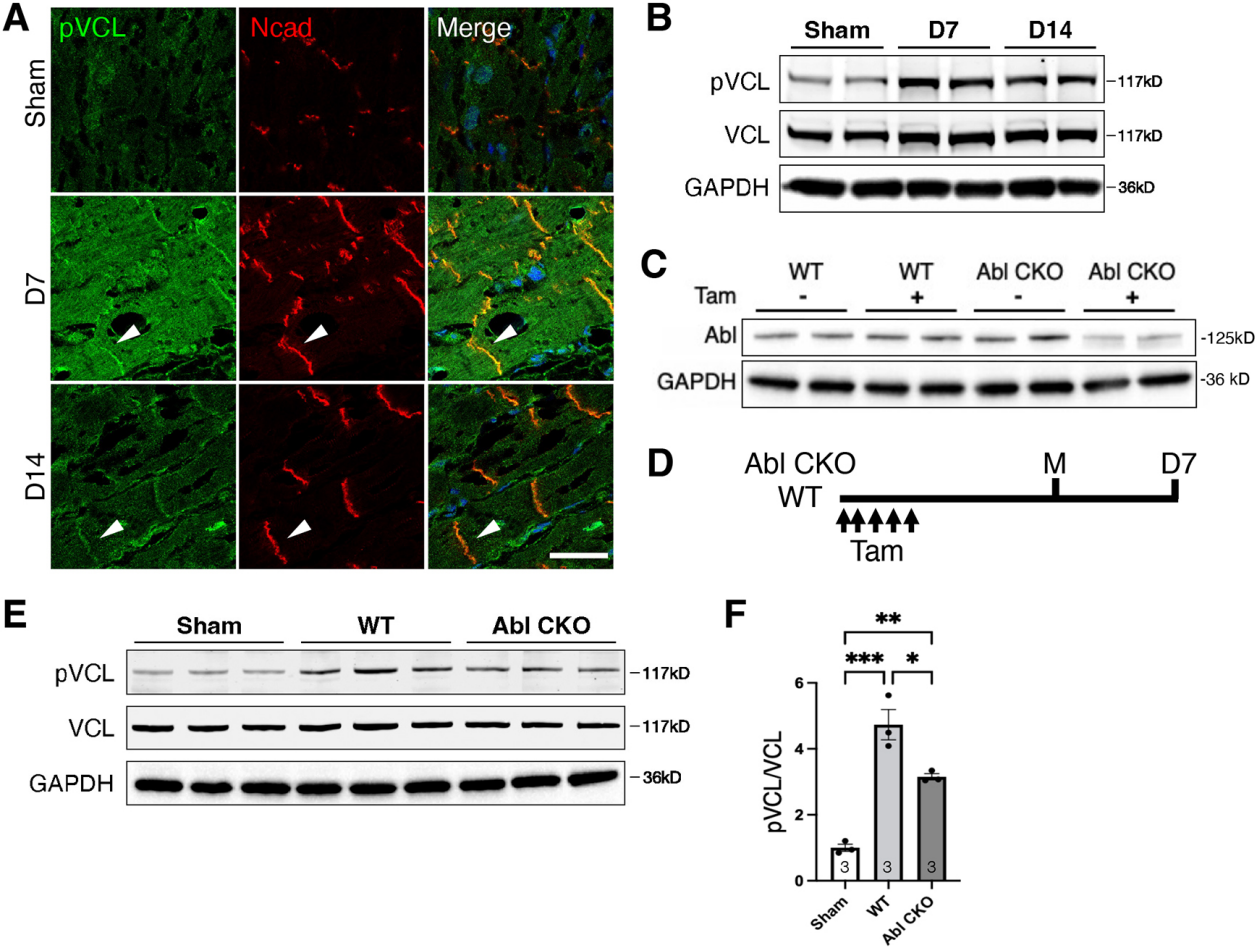
**Increased VCL pY822 in post-MI hearts requires ABL kinase.** (A) Representative immunofluorescence images of sham-operated (Sham), day 7 (D7), and D14 post-MI border zone (BZ) heart sections from WT rats stained for VCL pY822 (pVCL, green), N-cadherin (Ncad, red), and nuclei (blue). Individual pVCL and Ncad channels are shown along with the merged image. Arrowheads indicate colocalization of pVCL and N-cadherin at the ICD. Images representative of three independent experiments. Scale bar: 50 µm. (B) Immunoblots of BZ lysates from Sham, D7, and D14 rat hearts probed for pY822 VCL (pVCL), total VCL, and GAPDH. (C) Immunoblots of WT and Abl CKO (Abl f/f; MHC-MerCreMer) mouse hearts with (+) or without (−) tamoxifen (Tam) treatment. Blots probed for ABL and GAPDH. (D) Scheme for Tam-induced depletion of ABL in adult mice before MI. Blots in B and C representative of two independent experiments. (E,F) Immunoblots (E) and quantitative analysis (F) of MI BZ lysates from WT and Abl CKO mice at day 7 post-MI (*n*=3 mice per condition). Left ventricular heart lysate from sham-operated animals was used as a control. *P<0.05, **P<0.01, ***P<0.001 (one-way ANOVA with Tukey's multiple comparisons). Error bars represent s.d.

We questioned whether promoting adhesion remodeling in mature hearts would increase VCL pY822 expression and recruitment to adhesions. Following a heart attack, CM cell–cell and cell–ECM interactions remodel in the border zone (BZ) of the infarcted myocardium (Matsushita et al., 1999; van Dijk et al., 2008) to resemble those seen in the neonatal heart (Angst et al., 1997; Hanson et al., 2013; Hirschy et al., 2006; Hortells et al., 2019). To investigate whether VCL pY822 expression changes in response to injury, we used a myocardial infarction (MI) model to induce ischemic injury in the adult heart. Two-month-old Sprague Dawley rats underwent left anterior descending (LAD) coronary artery ligation, and the BZ was surgically resected away from the infarct zone for western blot analysis. We found that VCL pY822 levels were elevated in the MI group compared to sham-operated controls (Fig. 2A,B). Notably, VCL pY822 immunostaining revealed strong enrichment at the ICD as well as increased cytoplasmic expression in post-MI heart sections (Fig. 2A; Fig. S1). Therefore, we observed an upregulation of cardiac VCL pY822 in two contexts associated with dynamic adhesions – development and response to acute injury.

Previous *in vitro* studies have shown that ABL kinase regulates phosphorylation at VCL Y822 (Bays et al., 2014; Bertocchi et al., 2017). To determine whether ABL is responsible for the increased VCL pY822 post-MI, we generated a tamoxifen (Tam)-inducible cardiac-restricted *Abl* conditional knockout (*Abl* CKO) model by breeding *Abl1* flox/flox and αMHC-MerCreMer mice (Moresco et al., 2005; Sohal et al., 2001). At 2 weeks after Tam treatment, loss of ABL was confirmed in *Abl* CKO hearts by immunoblotting (Fig. 2C). Note that ABL is ubiquitously expressed; hence, the remaining ABL protein is likely from non-myocytes. Next, following Tam treatment, MI surgery was performed on 10-week-old WT and *Abl* CKO mice, and heart tissue was collected 7 days later (Fig. 2D). We found that the increased VCL pY822 detected in control BZ CMs was blunted in *Abl* CKO hearts post-MI (Fig. 2E,F), demonstrating that ABL kinase is at least partially responsible for VCL Y822 phosphorylation following tissue injury.

### *Vcl Y822F* mutant mice are viable

**Fig. 3.**
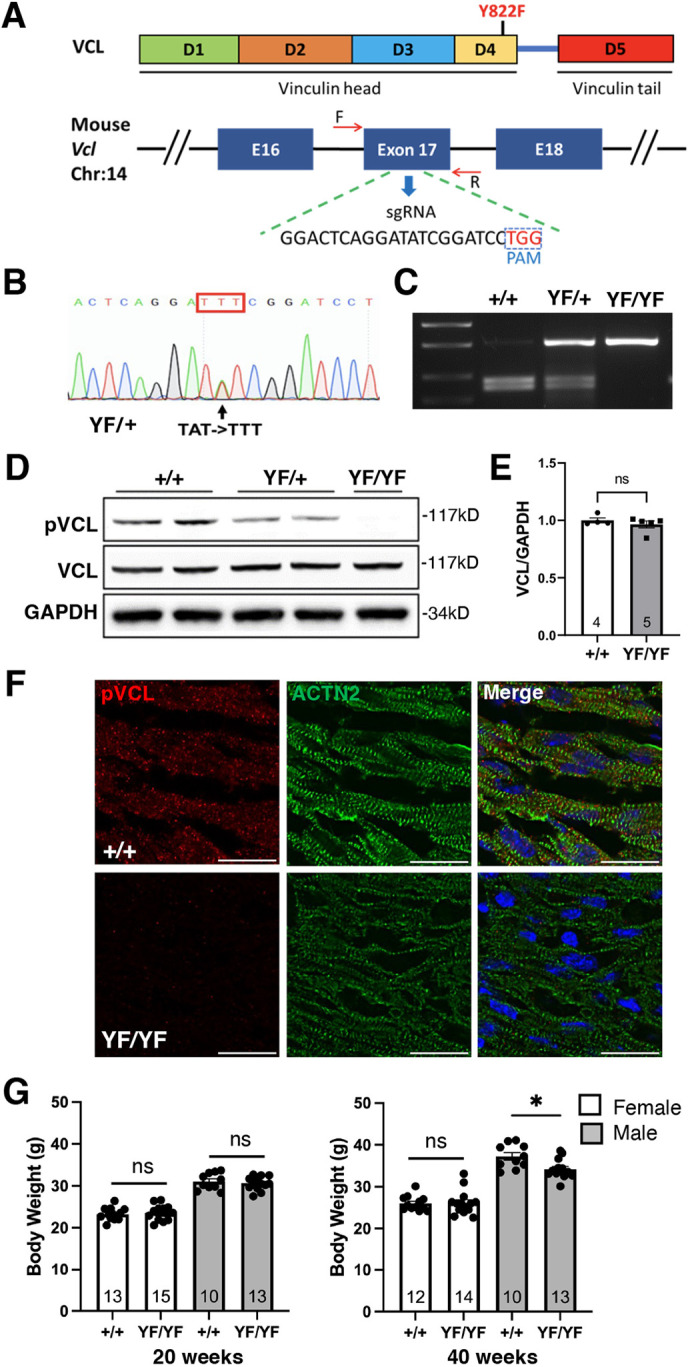
**Generation of *Vcl Y822F* mutant mice.** (A) Schematic of VCL protein structure (top) indicating the location of the Y822F mutation and the *Vcl* gene locus (bottom) showing the exon 17 sequence targeted by the sgRNA. (B) Sanger sequencing of the nucleotide change confirmed the *Vcl Y822F* (YF) mutation in a heterozygous *Vcl Y822* mutant mouse (YF/+). (C) PCR genotyping of WT (+/+), YF/+, and homozygous mutant (YF/YF) mice. The mutation destroyed an EcoRV site (GA**TAT**C>GA**TTT**C) in exon 17 of the *Vcl* gene. The PCR product was digested with EcoRV, resulting in two WT bands or an undigested full-length band corresponding to the YF mutation. (D) Immunoblots of WT, YF/+, and YF/YF P4 heart lysates. (E) Quantification of total VCL protein levels in WT (*n*=4) and Vcl Y822F (*n*=5) P4 heart lysates. Error bars are s.e.m. (F) Representative immunofluorescence images of heart sections from WT and *Vcl Y822F* P1 mice stained for VCL pY822 (pVCL, red), α-actinin (ACTN2, green) and nuclei (blue). Individual pVCL and ACTN2 channels are shown along with the merged image. Images representative of three independent experiments. Scale bar: 25 µm. (G) Body weight of female and male WT and YF/YF mice at 20 and 40 weeks. The number of mice in each group is listed in the column. *P=0.0105; ns, not significant (two-tailed unpaired Student's *t*-test).

To investigate the function of VCL pY822 *in vivo*, we created a new strain of *Vcl* knock-in mice. *Vcl Y822* was mutated to a non-phosphorylatable phenylalanine residue using CRISPR/Cas9-mediated gene editing to generate *Vcl^Y822F/+^* mice (Fig. 3A–C). *Vcl^Y822F/Y822F^* animals were born at the expected Mendelian frequency from *Vcl^Y822F/+^* intercrosses (53 *Vcl^+/+^*; 136 *Vcl^Y822F/+^*; 65 *Vcl^Y822F/Y822F^*, *n*=254). Loss of VCL pY822 in the *Vcl* mutant hearts was confirmed by immunoblotting and immunostaining (Fig. 3D,F). Importantly, immunoblot analysis of heart lysates revealed that total VCL protein levels in *Vcl Y822F* mice were similar to those in control animals (Fig. 3E), indicating that the Y822F mutation does not disrupt VCL stability. Next, we examined the localization of VCL pY822 in P1 heart sections. The punctate pY822 staining pattern observed in control hearts was lost in *Vcl* mutant CMs (Fig. 3F). Together, these results showed that we successfully created a viable VCL pY822-deficient mouse. Notably, *Vcl Y822F* mice displayed no obvious macroscopic phenotypic abnormality. A link between cell mechanics and metabolism (Dawson et al., 2023; Evers et al., 2021) led us to examine the body mass of the animals. We found a decrease in body weight (<10%) between *Vcl^+/+^* and *Vcl^Y822F/Y822F^* male mice, but not in female mice, at 40 weeks (Fig. 3G). Future work will be necessary to elucidate a possible sex-specific metabolic defect in *Vcl Y822F* mice.

### Loss of VCL pY822 alters cell–ECM and cell–cell adhesions in the postnatal heart

After birth, CMs elongate and myofibrils align during maturation, resulting in rod-shaped, terminally differentiated CMs (Angst et al., 1997). During this developmental progression, AJs and FAs are initially distributed all along the cell border. However, as CMs mature, FAs localize along the lateral membrane, and AJs become restricted to the bipolar ends of CMs (Hirschy et al., 2006). Notably, profound changes in the surrounding ECM accompany CM adhesion reorganization and maturation (Hanson et al., 2013). During embryonic development, fibronectin (FN) is the primary cardiac ECM protein. However, after birth, FN declines, and Type I collagen (COL) and laminin increase along the lateral membrane, resulting in stiffer cardiac tissue (Hanson et al., 2013).

**Fig. 4.**
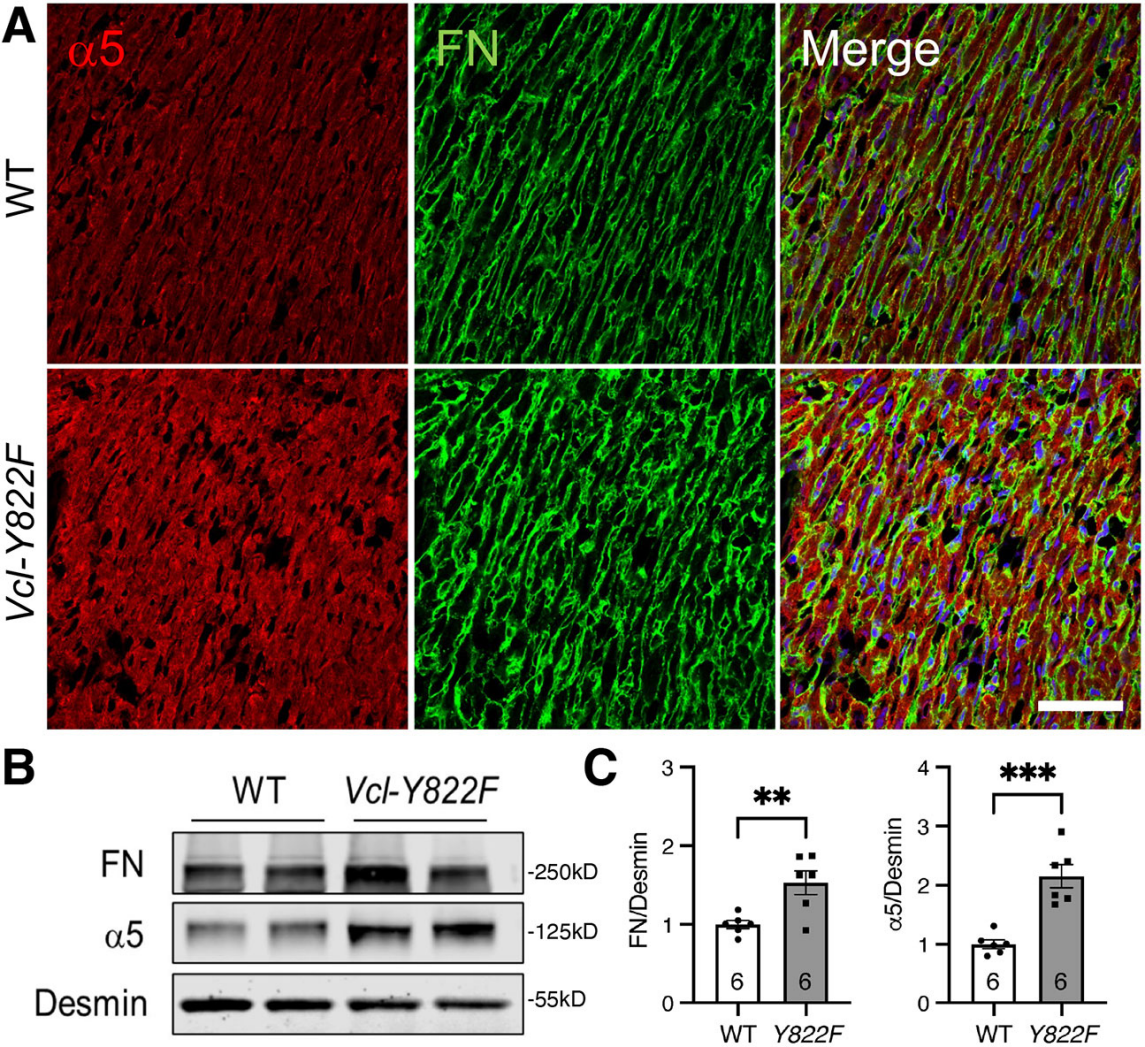
**Increased cell–ECM adhesions in *Vcl Y822F* hearts.** (A) Representative immunofluorescence images of P7 heart sections from WT and *Vcl Y822F* mice stained for α5 integrin (α5, red), fibronectin (FN, green) and nuclei (blue). Individual α5 and FN channels are shown along with the merged image. Images representative of three independent experiments. Scale bar: 50 µm. (B) Immunoblots of DOC-insoluble P7 heart lysates from WT and *Vcl Y822F* mice probed for FN, α5 and desmin. (C) Quantification of FN/desmin and α5/desmin ratios in WT and *Vcl Y822F* P7 heart lysates (*n*=6 mice of each genotype). **P<0.01; ***P<0.001 (two-tailed, unpaired Student's *t*-test). Error bars represent s.e.m.

As VCL functions at both AJs and FAs, we questioned whether the loss of Y822 affected (1) FA and ECM protein expression and organization, and (2) AJ protein expression and distribution. Given that VCL pY822 levels are highest in heart stages when FN is the primary ECM protein, we began by examining FA protein expression in P7 hearts. We analyzed the expression of FN and α5 integrin, a core component of the primary receptor for FN, in *Vcl Y822F* mutant and WT control hearts. Immunostaining of P7 heart sections revealed a marked increase in α5 integrin and FN expression in *Vcl Y822F* mutants compared to controls (Fig. 4A). Integrin–FN binding promotes the formation of FN fibrils that are initially soluble in the detergent deoxycholate (DOC) but are gradually converted into a stable, DOC-insoluble form that comprises the mature matrix (Sechler et al., 1996). Both α5 integrin and FN levels increased in the DOC-insoluble fraction at P7, consistent with the increased lateralization of mutant VCL and suggestive of strengthened myocardial cell–matrix interactions in the *Vcl Y822F* hearts (Fig. 4B,C). Notably, there was no change in α5 integrin and FN levels at P14 and P28 (Fig. S2), suggesting the effects of VCL Y822F on cell–ECM adhesions are limited to the developmental window after birth when FN levels are high (Konstandin et al., 2013).

**Fig. 5.**
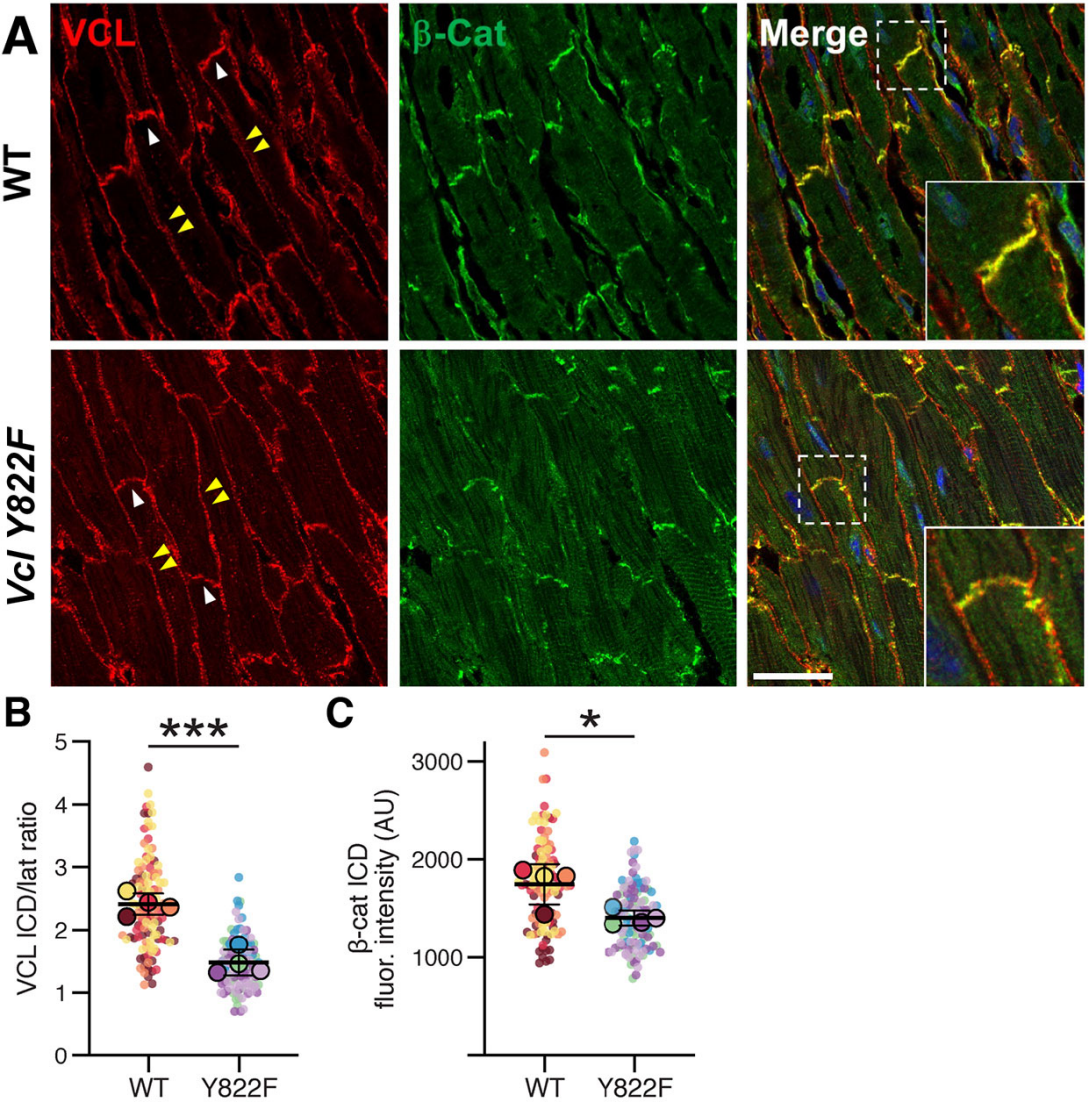
**Loss of VCL pY822 disrupts VCL distribution and AJ composition.** (A) Representative immunofluorescence images of P28 heart sections from WT and *Vcl Y822F* mice stained for VCL (red), β-catenin (β-Cat, green) and nuclei (blue). Individual VCL and β-Cat channels are shown, along with the merged image. White arrowheads mark ICDs, and double yellow arrowheads point to lateral membranes in the VCL image. The inset in the merged image is a high-magnification view of the boxed region, highlighting VCL recruitment to the ICD. Scale bar: 25 µm. (B) VCL ICD to lateral membrane fluorescence ratio in WT and *Vcl Y822F* P28 heart CMs. Data are presented as a SuperPlot, where individual measured CM membrane fluorescence ratios (data points) from one heart (biological replicate) are plotted as circles, and each heart is assigned a unique color (*n*=4 hearts per genotype). The mean ICD/lateral fluorescence ratio from each heart is overlaid on the individual data points as a larger, color-matched circle outlined in black. The mean±s.d. of the four hearts are shown as black bars. (C) β-catenin fluorescence intensity at CM ICDs was measured in four WT and four *Vcl Y822F* P28 heart sections. Data are presented as a Superplot as in B. *P<0.05; ***P<0.001 (unpaired two-tailed Welch's *t*-test).

Next, we sought to determine whether the loss of VCL pY822 impacted AJ organization at ICDs. As the ICD is still developing at early postnatal stages (e.g. P7 and P14), we examined AJ composition in more mature P28 hearts. We stained heart sections for VCL and β-catenin and quantified protein recruitment to ICDs. (Fig. 5A–C). VCL was recruited to both CM termini (ICDs) and lateral membranes in WT and Vcl Y822F hearts. However, the VCL ICD-to-lateral fluorescence intensity ratio was significantly lower in *Vcl Y822F* CMs compared to that seen in WT (Fig. 5B). β-catenin intensity was weaker at *Vcl Y822F* CM termini (Fig. 5C), suggesting a reduction in AJs compared to in WT. Moreover, VCL and β-catenin appeared punctate at the ICD in *Vcl Y822F* mutants compared to the linear pattern observed in WT hearts (Fig. 5A, inset). Next, we examined the expression of the N-cadherin–catenin complex, the protein core of the AJ, and associated proteins in heart lysates from P14 and P28 mice (Fig. S3). There was no change in N-cadherin, αE-catenin, αT-catenin, β-catenin and afadin protein levels between WT and *Vcl Y822F* hearts (Fig. S3). Finally, we measured fetal gene expression in WT and *Vcl Y822F* hearts to determine whether the loss of pY822 affected CM maturation. Quantitative RT-PCR revealed no change in the expression of fetal genes *Nppa*, *Nppb*, *Myh7* or the adult myosin isoform *Myh6*, in *Vcl Y822F* hearts compared to in WT controls, consistent with normal CM differentiation (Fig. S4). Collectively, these results show that VCL Y822 phosphorylation regulates cell–ECM and cell–cell adhesions during postnatal heart development.

As loss of pY822 affected both cell–ECM and cell–cell adhesions, we questioned whether other VCL regulatory pathways were altered in *Vcl Y822F* mice. For example, phosphorylation of VCL Y100 and Y0165 by SRC kinase regulates VCL protein dynamics at FAs (Auernheimer et al., 2015; Zhang et al., 2004). We examined VCL pY100 and pY1065 levels in WT and *Vcl Y822F* hearts using phospho-specific antibodies. We observed no difference in pY100 or pY1065 levels in *Vcl Y822F* P7 heart lysates compared to the control (Fig. S5), suggesting that SRC-mediated phosphorylation of VCL is not perturbed in *Vcl Y822F* mice.

### pY822 regulates VCL recruitment to cell–cell contacts in cultured neonatal CMs

**Fig. 6.**
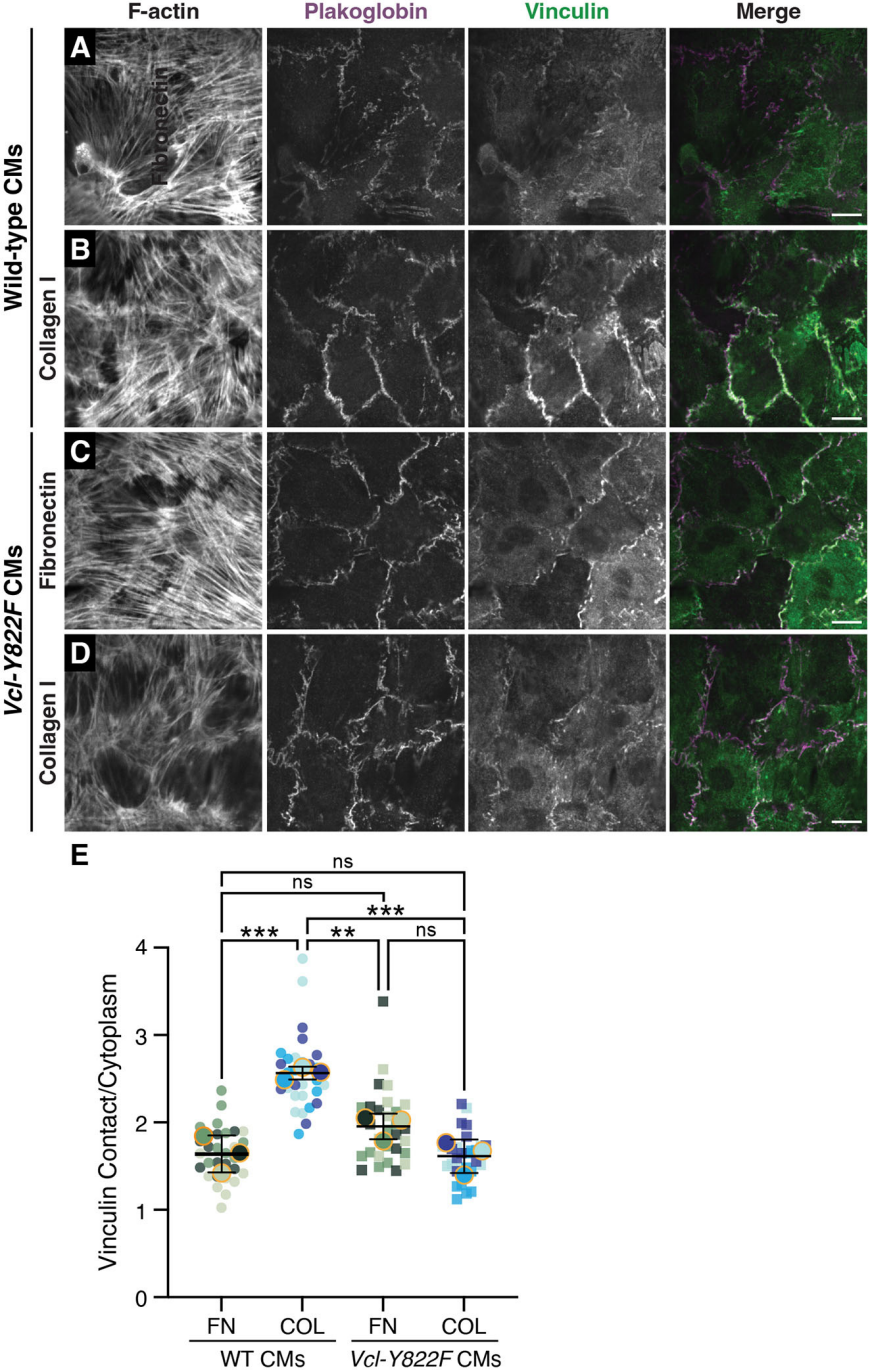
**Y822 phosphorylation regulates VCL recruitment to cell-cell contacts in cultured CMs.** (A–D) Wild-type (A,B) or *Vcl Y822F* CMs (C,D) plated on fibronectin (FN) or collagen I (COL)-coated PDMS, fixed 72 h post-plating, and stained for F-actin, plakoglobin and VCL. (E) VCL contact to cytoplasmic fluorescence ratio from cells imaged in A–D was measured from three biological replicates. Data are presented as a Superplot, where each image is plotted as a circle (WT cells) or square (*Y822F* cells) and colored (shades of green for cells plates on FN; shades of blue for cells plated on COL I) to identify samples in each replicate. The mean fluorescence ratio of each replicate is overlaid on the individual data points as a larger, color-matched circle outlined in orange. The mean±s.d. of the three replicates are shown as black bars. **P<0.01; ***P<0.001 (one-way ANOVA with Tukey's multiple comparisons). Scale bars: 10 µm.

CM maturation correlates with a shift in the heart ECM, from FN to COL. To determine whether pY822 regulates ECM-dependent VCL enrichment at adhesions, we isolated CMs from homozygous mutant *Vcl Y822F* neonates (isogenic C57BL/6J) and CMs isolated from C57BL/6J mice (referred to as wild type, WT). We plated CMs on FN and COL-coated PDMS substrates and stained them for VCL and plakoglobin, a cadherin-binding protein, to determine VCL recruitment to cell–cell contacts. We measured similar levels of VCL recruitment to cell–cell adhesions in WT and *Vcl Y822F* CMs on FN (Fig. 6A–E). Notably, we observed a significant increase in VCL recruitment to cell–cell contacts in WT CMs plated on COL versus FN (Fig. 6A,B,E). Strikingly, VCL enrichment at cell–cell contacts on COL was lost in *Y822F* CMs (Fig. 6C–E). Given that COL is the primary matrix protein in P28 hearts (Hortells et al., 2019), the reduction in VCL recruitment to cell–cell contacts in *Vcl* Y822F CM cultures is reminiscent of the defect in VCL adhesion distribution observed in *Vcl Y822F* hearts (Fig. 5).

### VCL Y822F dynamics are similar to VCL WT

**Fig. 7.**
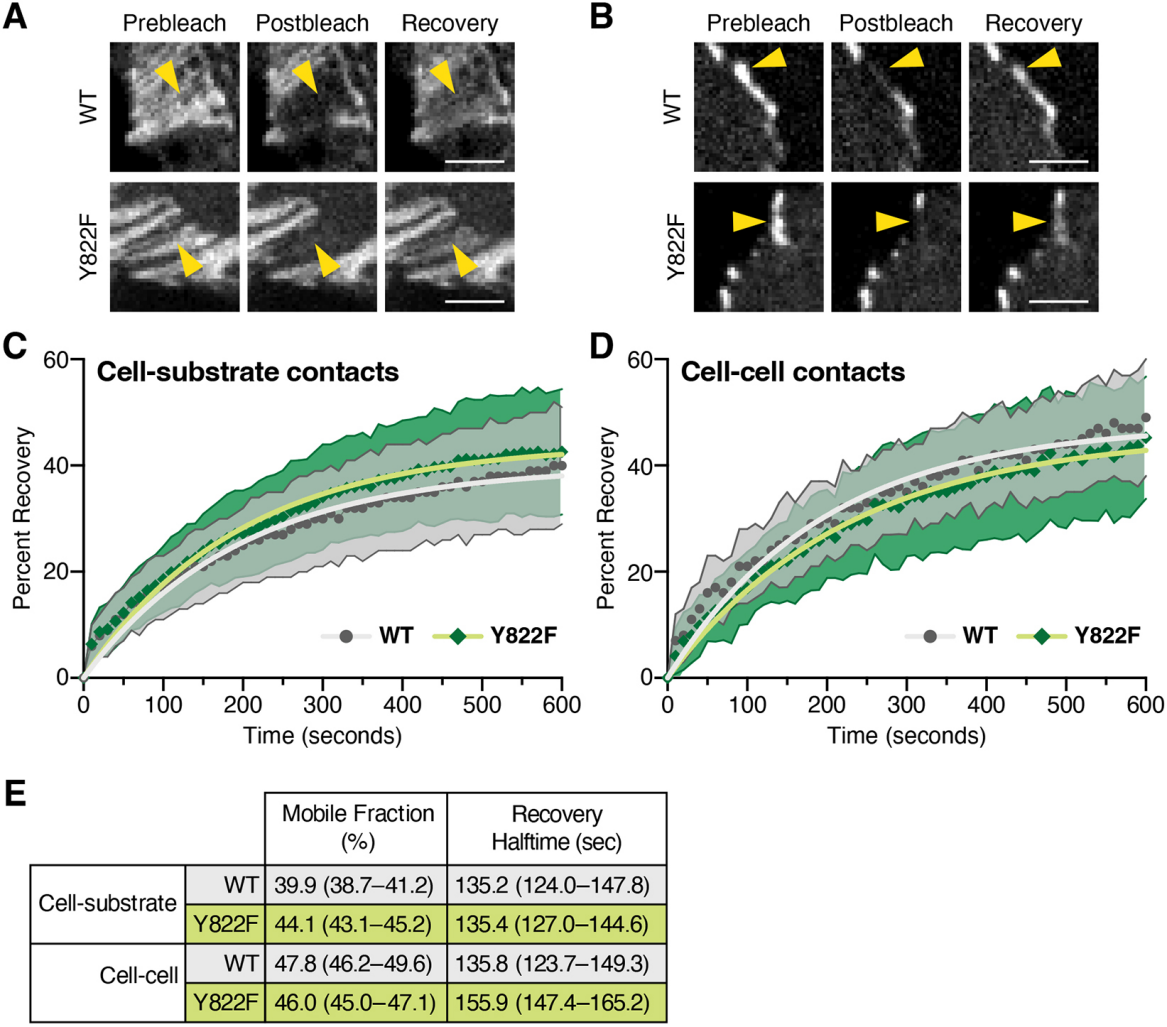
**VCL Y822F dynamics are similar to VCL WT.** (A,B) Images from FRAP experiments showing pre-bleach, post-bleach and recovery after 10 min in WT CMs expressing Emerald-tagged VCL WT or *Vcl Y822F* CMs expressing VCL Y822F at cell–substrate contacts (A) or cell–cell contacts (B). Yellow arrowheads mark the FRAP region. Scale bars: 5 µm. (C,D) Plots of mean±s.d. of FRAP recovery fraction over time for Emerald–VCL WT expressed in WT CMs (WT, dark gray circles mark the mean; light gray shaded area defines the s.d.) and Emerald–VCL Y822F expressed in *Vcl Y822F* CMs (green diamonds mark the mean; green shaded area defines the s.d.). Recovery plots are shown for cell–substrate (C) and cell–cell (D) contacts. The data were fit to a single exponential curve (WT, light gray line; Y822F, light green line). The number of experimental replicates and FRAP regions quantified were: cell substrate WT (3, 46), Y822F (3, 39); cell–cell WT (3,36), Y822F (4, 42). (E) Summary of the mobile fraction and recovery halftimes with 95% confidence interval.

The reduced VCL recruitment to AJs in Y822F CMs, observed both *in vivo* and *in vitro,* led us to question whether the *Y822F* mutation impacts VCL dynamics at CM FAs and AJs. To test this, mEmerald (Em)-tagged VCL and VCL Y822F were individually transfected into WT or *Vcl Y822F* CMs, respectively, cultured on COL. As expected, Em–VCL localized to CM FAs and AJs (Fig. S6A). Em–VCL Y822F also localized to both FAs and AJs (Fig. S6A). However, we observed fewer and smaller VCL–Y822F-positive AJs in *Vcl* Y822F CMs (Fig. S6B–E). Protein dynamics were measured using fluorescent recovery after photobleaching (FRAP) in confluent CMs cultured for 48–72 h (Merkel et al., 2019). Fluorescence recovery at FAs and AJs over 10 min was quantified, plotted and fit to a single exponential curve (Fig. 7A–D). The mobile fractions of VCL and VCL Y822F were similar at both FAs and AJs, as indicated by the overlapping confidence intervals (Fig. 7E). We then assessed the recovery rates of VCL and VCL Y822F. The recovery halftimes of VCL and VCL Y822F were also similar at AJs and FAs, as indicated by the overlapping confidence intervals (Fig. 7E). We conclude that VCL dynamics at both junction types are (1) similar despite distinct binding partners and (2) the Y822F mutation does not affect protein dynamics at stable junctions. We propose that differences in VCL recruitment in the Y822F background might be due to differences in available binding sites for VCL Y822F at cell–cell contacts. However, further work is necessary to establish the underlying mechanism.

### *Vcl Y822F* mutant mice exhibit cardiac dysfunction

**Fig. 8.**
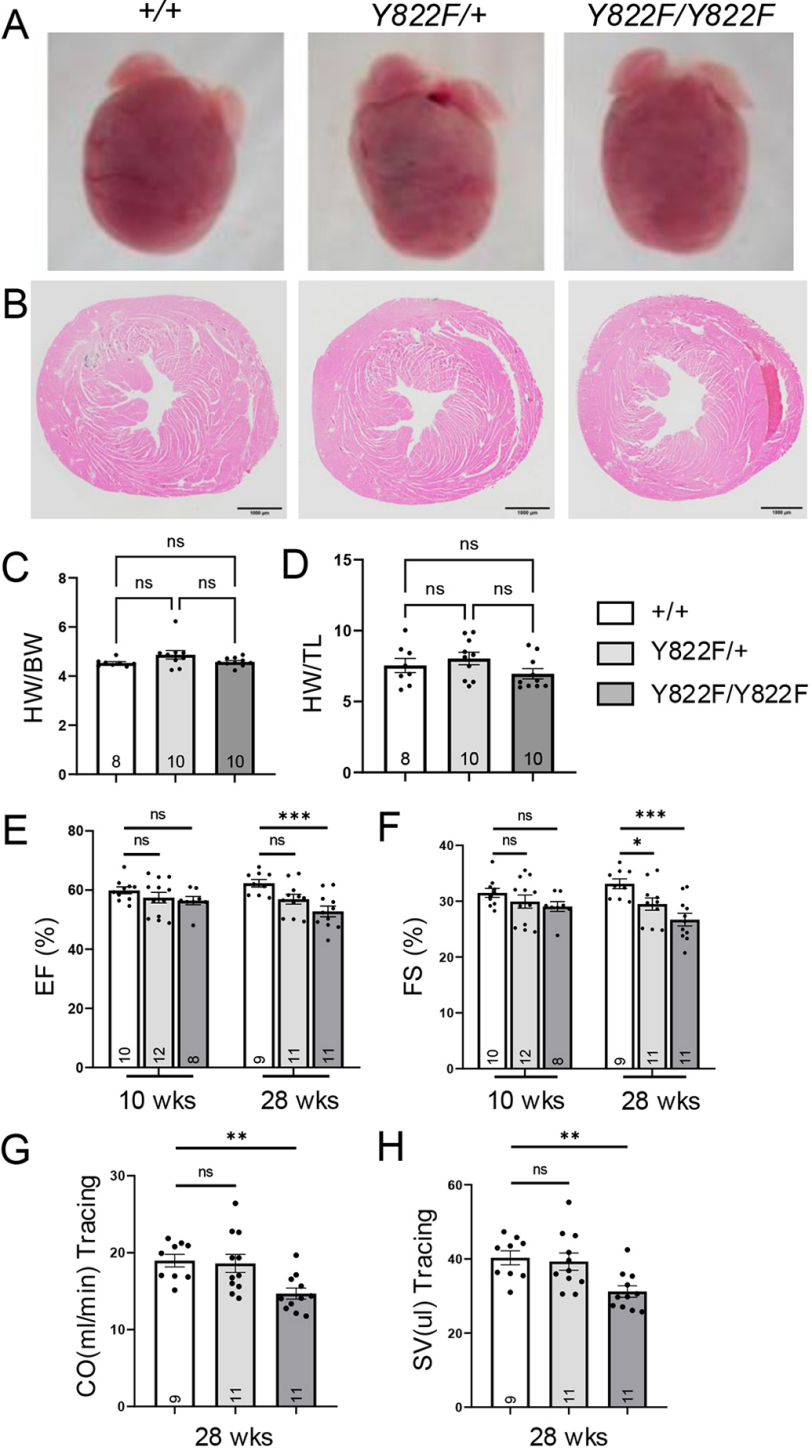
**Histological and cardiac function analysis of *Vcl Y822F* hearts.** (A) Representative whole-mount images of hearts from *Vcl +/+*, *Vcl Y822F/+,* and *Vcl Y822F/Y822F* mice at 40 weeks (wks) of age. (B) Representative H&E-stained heart sections from 28-week-old mice. (C,D) Quantification of heart weight/body weight ratio (C) and heart weight/tibia length ratio (D) in *Vcl +/+*, *Vcl Y822F/+*, and *Vcl Y822F/*Y822F mice at 40 weeks of age. The number of mice studied is listed in the column. ns, not significant (one-way ANOVA with Tukey's multiple comparisons). (E–H) Serial echocardiographic analyses were performed on mice from 10 to 28 weeks (wks) of age. Comparison of (E) ejection fraction (EF) and (F) fraction shortening (FS) at 10 and 28 weeks of age, and (G) cardiac output (CO) and (H) stroke volume (SV) at 28 weeks of age is shown. The number of mice studied is listed in the column. *P<0.05, **P<0.01, ***P<0.001 (one-way ANOVA with Dunnett's multiple comparison versus WT group). Error bars represent s.e.m.

To determine whether *Vcl Y822F* mutants exhibit a cardiac phenotype over time, we performed histological and functional analyses on adult mice. We did not detect any morphological or histological abnormalities in *Vcl Y822F* hearts at 28 weeks of age (Fig. 8A,B). Staining with Masson's Trichrome revealed no apparent fibrosis in the *Vcl* mutant hearts (Fig. S7). Moreover, we found no difference in heart weight-to-body weight ratio or heart weight-to-tibia length ratio between WT and *Vcl Y822F* mutant mice at 40 weeks of age (Fig. 8C,D). To assess cardiac function, we performed serial echocardiography on *Vcl* mutant mice at 10 and 28 weeks of age (Fig. 8E–H; Table S1). No difference in LV ejection fraction (EF) or fraction shortening (FS) was observed in the *Vcl Y822F* mice at 10 weeks of age. However, *Vcl* mutant mice exhibited a reduction in LV function at 28 weeks (Fig. 8E–H, Table S1), suggesting that adhesion defects caused by loss of pY822 in young hearts manifest as functional defects with age. These data provide the first *in vivo* evidence that VCL Y822 phosphorylation plays a role in cardiac function.

## DISCUSSION

VCL phosphorylation has emerged as a crucial post-translational regulator of adhesive activity at FAs and AJs in cultured cells (Bays and DeMali, 2017; Goldmann, 2016). Here, we provide evidence that phosphorylation regulates VCL function in the heart. We show that VCL pY822 levels in the mouse heart correlated with profound changes in adhesion dynamics and junctional remodeling in CMs, both during development and post MI. We created a new *Vcl Y822F* mutant mouse and found that VCL pY822 regulates both AJs and FAs in maturing CMs. Experiments in cultured CMs provided further support for the role of pY822 in regulating VCL function at AJs. Finally, *Vcl Y822F* mutant mice exhibited a cardiac defect with age, suggesting that loss of pY822 has downstream effects on heart function. Although future work is needed to investigate the role of VCL pY822 during heart development and post injury, this study provides new insights into the importance of phosphorylation in regulating adaptor function in different tissue environments.

### VCL Y822 phosphorylation peaks during adhesion remodeling in CMs

VCL pY822 levels were highest in the developing and perinatal heart but decreased significantly after 2 weeks of age and remained low in the adult heart. This expression pattern correlates with the sweeping rearrangement and realignment of cell adhesions during CM maturation that allow CMs to adapt to increased cardiac demand and growth. In the developing heart, VCL pY822 localization in CMs was punctate and cytoplasmic. Previously, VCL pY822 was found to colocalize with E-cadherin at AJs in confluent cultures of mammary epithelial cells (Bays et al., 2014). However, VCL pY822 has been reported to be largely punctate and cytoplasmic in other epithelial cell models in the absence of activation or stimulus (Birukova et al., 2016; Onochie et al., 2019).

Following a heart attack, CMs in the BZ of infarcted myocardium remodel AJs and FAs to resemble those seen in the neonatal heart (Angst et al., 1997; Matsushita et al., 1999). Notably, VCL pY822 was induced in adult BZ CMs in a rodent MI model. Robust VCL pY822 recruitment to CM AJs was only observed following acute injury. In developing CMs where pY822 levels are highest (E13.5–P1), emerging AJs and FAs are intermixed along the cell membrane. By contrast, in mature CMs where pY822 levels are lowest, established AJs and FAs are concentrated at the ICD and lateral membrane, respectively. Cardiac injury induces both adhesion remodeling and VCL Y822 phosphorylation in adult CMs; thus, VCL pY822 is enriched at remodeling AJs along the ICD. We conclude that the expression and cellular distribution of VCL pY822 depend on multiple factors – cell type, maturation state of the cadherin adhesion complex and cytoskeletal tension. Further work is needed to determine how pY822 regulates VCL localization and function.

### VCL pY822 function in the heart

To investigate the role of pY822 *in vivo*, we created a new *Vcl Y822F* mutant mouse using CRISPR/Cas9 genome editing. *Vcl Y822F* mice were viable, fertile and lacked gross morphological defects. By contrast, complete loss of VCL leads to mid-gestation lethality and numerous developmental abnormalities, including cardiac defects (Xu et al., 1998). Thus, Y822 phosphorylation is not essential for VCL function in embryogenesis and viability. Instead, our data suggest that pY822 plays an important role in regulating VCL function in CM maturation during postnatal heart development.

We observed increased α5 integrin and FN expression in Vcl Y822F neonatal (P7) hearts relative to WT controls, suggesting enhanced cell–ECM adhesion. We speculate that increased cell–ECM adhesion is a compensatory response to weakened cell–cell adhesion at this neonatal stage of heart development. However, the lack of well-formed ICDs at P7 prevented us from assessing AJ composition. The increase in α5 integrin and FN expression was transient, as no difference in expression was observed at later stages of heart development (P14 and P28) when the ECM transitions from FN to COL and the CM ICD matures.

We then documented AJ defects in older hearts (P28) with established, although still maturing, ICDs. Increased COL and decreased FN create a stiffer matrix in the P28 heart relative to the neonatal heart (Hortells et al., 2019). Loss of Y822 phosphorylation caused a significant shift in VCL distribution, as reflected in a reduced ICD-to-lateral membrane ratio, in P28 CMs. Alterations in AJ composition were also observed. However, AJ protein levels were not altered in *Vcl Y822F* hearts, consistent with a defect in protein organization rather than protein expression. Notably, VCL recruitment to AJs was reduced significantly in isolated *VCL Y822F* CMs cultured on COL, consistent with changes observed *in vivo*. Loss of VCL pY822 did not disrupt VCL recruitment to AJs in CMs cultured on FN, suggesting pY822 function is ECM specific. Future work is expected to determine how VCL pY822 is regulated in response to changes in ECM and how VCL pY822 functions in AJ organization and ICD maturation during heart development. As we observed changes in localization but not turnover rates between VCL WT and VCL Y822F, we postulate that a primary role of Y822 phosphorylation is the regulation of VCL-binding sites at AJs.

*Vcl Y822F* mice exhibited a significant reduction in LV function at 28 weeks, whereas younger mice (10 weeks) did not display a defect in heart function. We postulate that cardiac dysfunction in older *Vcl* mutant hearts results from compromised AJ organization during early stages of postnatal heart development that manifest later in life. Relatively modest changes in adhesion organization can have profound effects on heart function with age and/or stress [e.g. *PKP2* haploinsufficiency (Zhang et al., 2021)]. Further work is needed to determine how the heart function is compromised in VCL pY822-deficient mice.

Previously, VCL Y822 was shown to be phosphorylated by the non-receptor tyrosine kinase ABL in epithelial cells (Bays et al., 2014; Bertocchi et al., 2017). We used an *Abl* CKO mouse model to demonstrate that ABL kinase mediates Y822 phosphorylation in post-MI heart BZ CMs. Notably, BZ CMs have been shown to increase non-muscle myosin contractility (Pandey et al., 2018). We previously reported that activation of non-muscle myosin contractility promoted VCL Y822 phosphorylation and recruitment to CM cell–cell contacts in the neonatal heart (Li et al., 2023). We speculate that increased non-muscle myosin activity and enhanced VCL pY822 are required for CM adhesion remodeling post MI. Future work will use the *Vcl* Y822F mutant mouse to define the role of VCL pY822 in the post-MI heart and determine how non-muscle myosin activity regulates VCL Y822 phosphorylation and function during CM remodeling.

### VCL phosphoregulation beyond Y822

VCL phosphorylation is involved in various cellular processes. SRC kinase-mediated phosphorylation of VCL (at Y100 and Y1065) is important for platelet activation (Zhang et al., 2004). In airway smooth muscle, Y1065 phosphorylation creates an ‘open’, active VCL conformation that promotes actin polymerization in response to contractile stimulation (Huang et al., 2014). During collective cell migration of MDCK cells, S1033 phosphorylation biases VCL toward a closed, unloaded state at AJs (Shoyer et al., 2023). A phosphoproteomic study identified a new VCL phosphorylation site, serine 721 (pS721), associated with a porcine atherosclerosis model (Shih et al., 2023). In addition, a VCL S721C mutation was recently identified in an individual with dilated cardiomyopathy (ClinVar VCV003706424.1). Hence, VCL phosphorylation might be a widely used mechanism for responding to changes in mechanical forces, which are critical for heart health.

### Limitations

The *Vcl Y822F* mutant mouse described here is a constitutive knockout, preventing Y822 phosphorylation in all tissues. Thus, the *Y822F* mutation could affect VCL function in other cardiac or vascular cell types (e.g. cardiac fibroblasts and endothelial cells). In the future, a conditional *Vcl Y822F* mutant mouse could address cell autonomous, tissue- and developmental stage-specific functions of VCL pY822 in heart development and disease.

### Conclusion

Our study provides evidence that phosphorylation regulates VCL function in adhesion organization both *in vivo* and *in vitro*. Collectively, our data support a model in which VCL pY822 facilitates the dynamic remodeling of CM adhesions required for CM maturation in the developing heart and response to injury in the adult heart. Adaptor proteins, such as VCL, are common phosphoproteins; however, the mechanism by which phosphorylation regulates protein function remains largely unknown. We propose that phosphorylation plays a key and previously unappreciated role in regulating adaptor proteins during periods of dynamic adhesion remodeling.

## MATERIALS AND METHODS

### Mouse model generation by CRISPR-mediated gene editing, genotyping, and colony maintenance

CRISPR-targeting reagents were injected into the cytoplasm of the C57BL/6J mouse zygotes by the Brown University Mouse Transgenic and Gene-Targeting Facility. The injection mix contained 100 ng µl^−l^ SpCas9 nuclease [Cat. No. 1081060, Integrated DNA Technologies (IDT)], 150 ng µl^−l^ the annealed guide RNA (crRNA:tracrRNA, IDT), and 250 ng µl^−l^ single-stranded DNA oligonucleotide carrying the targeted mutation *Vcl^Y822F^* (TAT>TtT) (IDT) and a silent mutation to disrupt the targeting site PAM domain (Table S2). A gRNA sequence close to the mutation site and with minimum potential off-target cleavage activity was selected (Table S3). Mosaic *Vcl^Y822F/+^* founder mice were bred with wild-type C57BL/6J mice for four generations to diminish potential off-target mutations. Two independent *Vcl^Y822F/+^* founder lines were intercrossed to generate homozygous *Vcl^Y822F/Y822F^* mice. Mice were genotyped by *Vcl* gene locus-specific PCR. The *Y822F* mutant allele resulted in the loss of an EcoRV site at the *Vcl* locus (GATATC>GAT**T**TC). Following EcoRV digestion of the PCR product (514 bp), the *Vcl^+/+^* genotype corresponds to two DNA bands (273 and 241 bps), *Vcl^Y822F/+^* has the full-length DNA band (514 bps) plus the two bands, and *Vcl^Y822F/V822F^* has only the undigested PCR product. To ensure inclusion of both sexes for these studies, the sex of neonatal pups was confirmed by the presence or absence of the Y chromosome via PCR and/or observation of the anogenital distance. All animal studies were performed in accordance with the guidelines of the IACUC of Brown University Health Rhode Island Hospital.

### Echocardiographic and hemodynamic measurements

Transthoracic two-dimensional echocardiography was performed on *Vcl^+/+^*, *Vcl^Y822F/+^*, and *Vcl^Y822F/Y822F^* mice with a Vevo 2100 ultrasound system equipped with a 30-MHz transducer (FUJIFILM VisualSonics Inc., Toronto, ON, Canada). Heart rate (HR), ejection fraction (EF%), fractional shortening (FS%), left ventricular internal dimensions (LVID), left ventricular volume (LV vol), LV posterior wall thickness (LVPW) and LV anterior wall thickness (LVPW) were measured from M-mode images at the plane bisecting the papillary muscles. LV dimensions were obtained from endocardial border tracing of end-systolic and end-diastolic frames. The researcher doing these analyses was unaware of the genotype of the mice.

### Surgical induction of myocardial ischemia injury

Mice at 10 weeks of age were used for myocardial infarction (MI) experiments in which left anterior descending (LAD) coronary artery ligation was performed as described previously (Reichert et al., 2017). Two-month-old rats subjected to LAD coronary artery ligation and sham-operated controls were purchased from Charles River Laboratories (Wilmington, MA, USA).

### Histological analysis

Mice hearts were isolated and washed in PBS. The hearts were then fixed in fresh 4% paraformaldehyde (PFA) at 4°C overnight, dehydrated, and embedded in paraffin. Longitudinal 5 μm deparaffinized sections were stained with Hematoxylin and Eosin (H&E; Poly Scientific R&D) or Masson Trichrome (Sigma), mounted, and imaged with an Olympus BX43 microscope system. Images were processed with cellSens™ Imaging Software.

### Immunofluorescence

Heart tissues were harvested and embedded in OCT (Sakura Finetek). OCT sections (6 µm) were fixed in 4% PFA for 10 min and then washed with PBS three times. Heart sections were then permeabilized in 0.1% Triton X-100 (in PBS) for 10 min at room temperature. Sections were blocked in T-BSA (5% BSA and 0.01% Triton X-100 in PBS) for 1 h at room temperature and incubated overnight at 4°C with primary antibodies against: N-cadherin (1:400, 610921, BD Bioscience), α5 integrin (1:200, 553319, BD bioscience), fibronectin (1:100, PA1-23693, Thermo Fisher Scientific), pY822 VCL (pVCL) (1:200, ab61071 or ab200825, Abcam; or 1:200, 44-1080G, Invitrogen), VCL (1:100, V4505 or V9131, Sigma), α-actinin (1:200, A7732, Sigma) and β-catenin (1:200, C2206, Sigma). Note that two different monoclonal antibodies (mAbs) against VCL did not recognize the VCL pY822 population. Similarly, a mAb against VCL has been reported not to recognize VCL pY1065 (Zhang et al., 2004), suggesting the altered conformation of phosphorylated VCL is not recognized by the VCL mAbs. After washing in PBS, sections were incubated with secondary antibody [Alexa Fluor (AF) 488-conjugated goat anti-rabbit or AF488-conjugated goat anti-mouse IgG, AF555-conjugated goat anti-mouse IgG or donkey anti-rabbit IgG, AF586-conjugated donkey anti-rat IgG, 1:200, Life Technologies) for 1 h at room temperature. Finally, the tissue sections were washed in PBS and mounted with ProLong Gold Antifade Reagent containing DAPI (Life Technologies). Images were acquired using a Nikon A1R Confocal Microscope System. Images were processed and analyzed using Nikon NIS-Elements software and FIJI ImageJ 1.53 software.

### Western blot analysis

Harvested mouse heart tissues were homogenized in a modified RIPA buffer (50 mM Tris-HCl pH 7.5, 150 mM NaCl, 1 mM EDTA pH 8.0, 1% NP-40, 0.5% Na deoxycholate and 0.1% SDS) containing protease inhibitor and phosphatase inhibitor cocktails II and III (Roche Diagnostics). After rotating at 4°C for 2 h, the mixtures were centrifuged at 13,000 ***g*** for 15 min at 4°C. Primary antibodies used were against: α5 integrin (1:200, sc-166681, Santa Cruz), pY822 VCL (pVCL; 1:1000, ab61071/ab200825, Abcam; or 1:1000, 44-1080G, Invitrogen), VCL (1:1000, V4505, Sigma), N-cadherin (1:1000, 610921, BD Bioscience), GAPDH (1:3000, 6c5, RDI), afadin (1:2000, A0349, Sigma), fibronectin (1:3000, PA1-23693, Thermo Fisher Scientific), αE-catenin (1:50, 711200, Invitrogen), αT-catenin (1:100, 13974-1-AP, Proteintech), β-catenin (1:200, C2206, Sigma), pY100 VCL (1:1000, 44-2078G, Invitrogen) and pY1065 VCL (1:1000, 44-1074G, Invitrogen). To normalize signals, blots were also analyzed with the anti-GAPDH antibody, followed by IRDye 680 or IRDye 800CW-conjugated secondary antibody (1:15,000, LI-COR, Lincoln, NE, USA). Anti-mouse- or anti-rabbit-IgG conjugated to HRP secondary antibody (1:3000, Bio-Rad) were also used and developed by SuperSignal West Pico PLUS Chemiluminescent Substrate (Thermo Fisher Scientific, Rockford, IL, USA). Membranes were imaged using the Odyssey Infrared Imaging System (LI-COR) or ChemiDoc Imaging System (Bio-Rad, Hercules, CA, USA). Quantification was performed with FIJI ImageJ 1.53 software.

### Preparation of deoxycholate soluble and insoluble fractions

Deoxycholate (DOC) soluble and insoluble fractions of harvested heart tissues were prepared as previously reported (Wierzbicka-Patynowski et al., 2004). Briefly, fresh heart tissues were homogenized in DOC lysis buffer (20 mM Tris-HCl pH 8.8, 2 mM EDTA, 2 mM PMSF, 2 mM iodoacetic acid, 2 mM N-ethylmaleimide and 2% DOC) with protease inhibitors, and the lysates were centrifuged at 13,000 ***g***, 4°C for 15 min. The supernatants were saved as the DOC soluble fraction. The pellets were re-suspended in SDS solubilization buffer (Tris-HCl pH 8.8, 20 mM EDTA 2 mM, 2 mM PMSF, 2 mM iodoacetic acid, 2 mM N-ethylmaleimide and 1% SDS) with protease inhibitors. The protein concentration of DOC soluble fractions was measured by BCA assay. The DOC insoluble sample volume for SDS-PAGE is based on protein concentration in the corresponding DOC soluble fraction.

### RNA extraction and qRT-PCR

RNA was isolated from P28 hearts from WT (three male and three female) and *Vcl* Y822F mutant (three male and three female) mice. Total mRNA was extracted from heart tissue with TRIzol (Life Technologies) and precipitated with isopropyl alcohol. The concentration of isolated mRNA was determined by NanoDrop, followed by treatment with the TURBO DNA-free™ kit (Life Technologies) to remove contaminating DNA. cDNA was synthesized from 1 μg of total mRNA using the High-Capacity RNA-to-cDNA kit (Thermo Fisher Scientific). qRT-PCR was performed using the Power SYBR^®^ Green PCR Mastermix (Thermo Fisher Scientific). Specific primers designed for RT-PCR were: Nppa forward 5′-CAAGAACCTGCTAGACCACC-3′, Nppa reverse 5′-AGCTGTTGCAGCCTAGTCC-3′; Nppb forward 5′-GCGGCATGGATCTCCTGAAGG-3′, Nppb reverse 5′-CCCAGGCAGAGTCAGAAACTG-3′; Myh6 forward 5′-CCAATGAGTACC GCGTGAA-3′, Myh6 reverse 5′-ACAGTCATGCCGGGATGAT-3′; Myh7 forward 5′-ATGTGCCGGACCTTGGAA-3′, Myh7 reverse 5′-CCTCGGGTTAGCTGAGAGATCA-3′; Gapdh forward 5′-CATCTTCCAGGAGCGAGACC-3′, Gapdh reverse 5′-CTCGTGGTTCACACCCATCA-3′. All target gene PCR reactions were run in triplicate to calculate the average cycle threshold (Ct). Relative fold changes in gene expression were calculated using the ΔΔCt method. Briefly, the average target gene Ct was normalized to the Gapdh Ct to calculate the ΔCt. Next, the ΔΔCt for each *Vcl Y822F* sample was determined by subtracting the average WT target gene ΔCt from individual *Vcl Y822F* target gene ΔCt values. Fold change in gene expression was calculated using the formula 2(−ΔΔCt).

### Plasmids

mEmerald–Vinculin-23 was a gift from Addgene plasmid #54302 (deposited by Michael Davidson). mEmerald–vinculin Y822F was made by site-directed mutagenesis.

### CM isolation and culture

All animal work was approved by the University of Pittsburgh Animal Research Protection Office. *Vcl^Y822F/V822F^* mice were used to generate *Vcl* Y822F homozygous mutant CMs. The *Vcl^Y822F/V822F^* mutant allele was maintained on a C57BL/6J background; therefore, inbred wild-type B6 mice were used to create WT control CMs.

Neonatal mouse CMs were isolated as described previously (Ehler et al., 2013). Briefly, mouse pups were euthanized at P1, and their hearts were removed, cleaned, minced and digested overnight at 4°C in 20 mM 2,3-butanedione monoxime (BDM) and 0.0125% trypsin in HBSS. The following day, heart tissue was digested in 15 mg/ml collagenase and dispase (Roche) in Leibovitz medium with 20 mM BDM to create a single-cell suspension. Cells were pre-plated for 1.5 h in plating medium (65% high glucose DMEM, 19% M-199, 10% horse serum, 5% FBS and 1% penicillin-streptomycin) to remove fibroblasts and endothelial cells. CMs were plated on MatTek dishes (1.5×10^5^) or PDMS substrates in plating medium. At 16 h post plating, the plating medium was exchanged for maintenance media (78% high-glucose DMEM, 17% M-199, 4% horse serum, 1% penicillin-streptomycin, 1 μM AraC, and 1 μM isoproterenol).

MatTek dishes (35 mm dish with 10 mm microwell) were coated with rat tail type I collagen (Millipore) diluted to 0.5 μg/μl in PBS for 1 h at room temperature. The dishes were dried and treated with UV radiation for 1 h, and then washed with PBS, dried, and stored in the dark at room temperature until use. PDMS substrates were prepared by mixing 20 parts of silicone elastomer base with one part of curing agent (SYLGARD, Dow Corning, USA) and stirring. The mixture was degassed for 20 min in a vacuum chamber to remove air bubbles. 22×22 mm glass coverslips (Corning) were cleaned by sequentially rinsing in ethanol and water, then flaming to sterilize. Sterilized coverslips were spin-coated with PDMS (Laurell Technologies Corporation, Model WS-400B-6NPP/LITE/8K) at 5000 rpm for 1 min and then baked at 60°C for 2 h. PDMS-coated coverslips were washed in ethanol for 4 h, followed by water overnight. The next day, PDMS-coated coverslips were dried with N2 gas flow and then activated with oxygen plasma (Harrick Plasma Cleaner, PDC-001) for 3 min. Activated coverslips were treated with 2% (3-aminopropyl)triethoxysilane (APTES) dissolved in 95% ethanol for 20 min at 60°C. Coverslips were washed 10 times in ethanol, followed by 10 washes in distilled water. Immediately after surface activation and treatment, type I collagen (0.5 µg/µl, Millipore) or fibronectin (0.025 µg/µl, Santa Cruz Biotechnology) was added, and coverslips were incubated for 1 h at room temperature. ECM-coated coverslips were stored at 4°C until use.

### Cultured CM immunofluorescence and microscopy

Cultured CMs were processed for immunofluorescence as follows: cells were fixed in warmed (37°C) 4% electron microscopy (EM) grade paraformaldehyde in PBS (with Ca^2+^ and Mg^2+^ unless otherwise noted) and 0.12 M sucrose for 10 min and washed twice with PBS. Cells were permeabilized with 0.2% Triton X-100 in PBS for 5 min and washed twice with PBS. Cells were blocked in 10% BSA (Sigma) in PBS for 1 h at room temperature. Samples were incubated with primary antibodies in PBS+1% BSA for 1 h at room temperature, washed 2× in PBS, incubated with secondary antibodies in PBS+1% for 1 h at room temperature, washed 2× in PBS, and then mounted in Prolong Glass (Thermo Fisher Scientific). All samples were cured for at least 24 h before imaging. Primary antibodies used for CM immunostaining were against: anti-αE-catenin (1:100; Enzo Life Sciences ALX-804-101-C100), anti-plakoglobin (1:100; Cell Signaling 2309), anti-vinculin (1:800; Sigma-Aldrich V9131) and anti-N-cadherin (1:250; Invitrogen 99-3900). Secondary antibodies used were goat anti-mouse- or anti-rabbit-IgG conjugated to AF488, AF568 or AF647 (1:250; Invitrogen). F-actin was visualized using an Alexa Fluor dye conjugated to phalloidin (1:100, Thermo Fisher Scientific).

Stained CMs were imaged with a 100×1.49 NA objective on a Nikon Eclipse Ti inverted microscope outfitted with a Prairie swept field confocal system, Agilent monolithic laser launch, and Andor iXon3 camera using NIS-Elements (Nikon) imaging software. Expression and staining levels were adjusted for presentation purposes in Photoshop (Adobe). All individual channel levels were adjusted the same within each figure.

### Cultured CM image analysis

To quantify VCL recruitment to CM cell–cell contacts, a maximum intensity projection of six *Z*-stack slices containing cell–cell contacts was generated in ImageJ. IsoJ Dark thresholding was then performed on the projected image to create a mask of the plakoglobin channel, defining the region of analysis. The signal intensity of VCL was then measured within the masked region. Next, three random measurements of VCL signal intensity in the cell cytoplasm were collected and averaged. Finally, the ratio of VCL intensity within the mask was divided by the average cytoplasmic signal to normalize between samples and calculate the contact-to-cytoplasmic ratio. Data were plotted with Prism software (GraphPad). A one-way ANOVA with multiple comparisons was performed to determine significance. The plot displays data from at least ten images of CMs from three distinct CM preparations.

To quantify cell–cell and cell–substrate adhesion size in transfected CMs used for FRAP, the first frame (pre-bleach) from each movie was selected. Moments thresholding was used to create a mask of the GFP signal and define adhesions in ImageJ (NIH). Adhesion size was then measured by particle analysis (range 1 to 40 μm) in ImageJ. All data were plotted using Prism software (GraphPad). Statistical significance was determined using a Mann–Whitney test. The plots represent data from at least three separate transfections from two unique CM preps.

### FRAP experiments

FRAP experiments were performed on a Nikon swept-field confocal microscope (described above), outfitted with a Tokai Hit cell incubator and a Bruker miniscanner. Actively contracting cells were maintained at 37°C in a humidified, 5% CO_2_ environment. User-defined regions along cell–cell contacts were bleached with a 405 laser, and recovery images were collected every 10 s for 10 min. FRAP data were quantified in ImageJ (NIH). Individual recovery data were analyzed in Prism (GraphPad) using nonlinear regression (curve fit) with one-phase association subject to two constraints: the plateau must be between 0 and 1, and the *y*-intercept must be equal to 0. Following analysis, individual recovery plots were excluded if the r^2^ value was below 0.8. The calculated plateau was then compared to the average of the last four fluorescence measurements in the recovery. The FRAP measurement was excluded if the difference between the calculated plateau and the fluorescence average was greater than 0.25. The remaining individual recovery profiles were placed into Excel (Microsoft) to calculate the average and standard deviation. The FRAP average and standard deviation were then plotted in Prism (GraphPad) and fit to a one-phase association curve to determine the mobile fraction and half-time of recovery. Final FRAP recovery plots represent data from at least three separate transfections from two unique CM preps.

### Statistical analysis

Statistical differences were assessed in Prism (GraphPad) by unpaired two-tailed Student's *t*-test, unpaired two-tailed Welch's *t*-test, or one-way ANOVA followed by Tukey's or Dunnett's multiple comparison of individual means. SuperPlots were used to represent variability in larger datasets (Lord et al., 2020). A *P*<0.05 was considered statistically significant.

## Supplementary Material

10.1242/joces.263984_sup1 Supplementary information
