# Supplementary material for: Vinculin Y822 phosphorylation regulates adhesion remodeling during cardiomyocyte maturation
Source: J Cell Sci. 2025 Dec 18;138(24):jcs263984. doi: 10.1242/jcs.263984 (PMC12752503; doi:10.1242/jcs.263984)
Supplement: Supplementary information [file joces-138-263984-s1.pdf]

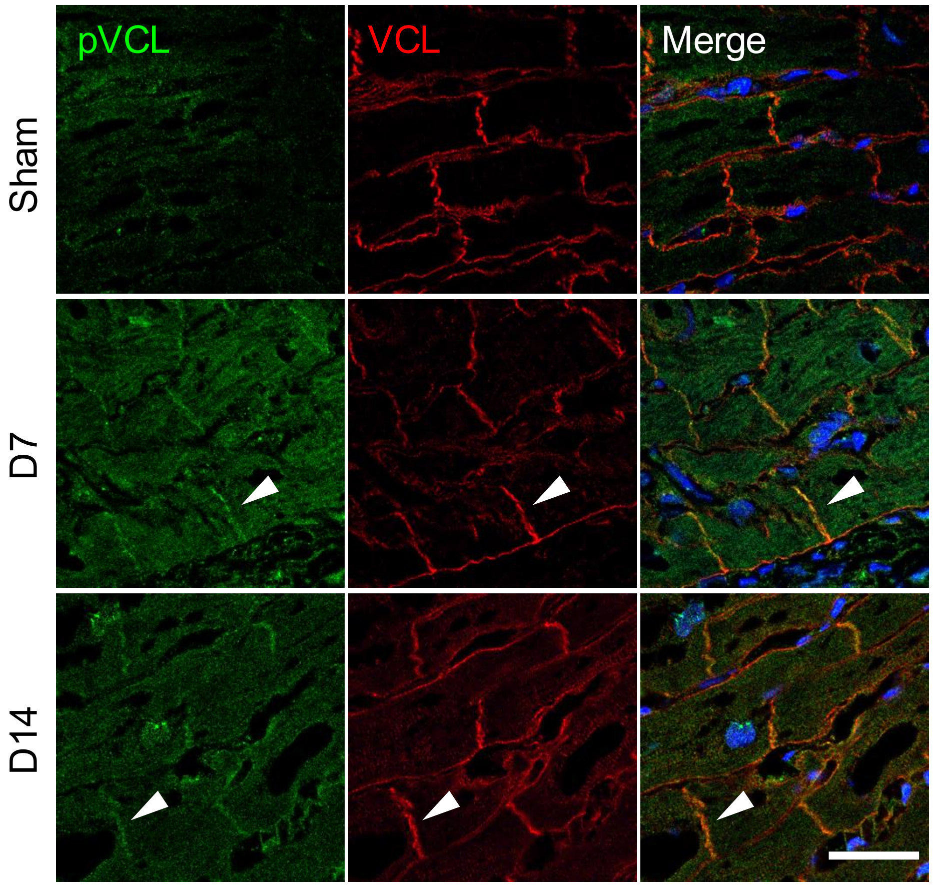

Fig. S1. Increased VCL pY822 in post-MI hearts. Representative immunofluorescence images of Sham, D7, and D14 post-MI BZ heart sections from WT rats stained for pY822 (pVCL, green), VCL (red), and nuclei (blue). Individual pVCL and VCL channels are shown along with the merged image. Arrowheads indicate co-localization of pVCL and VCL at the ICD. Scale bar: 50 μm.

Journal of Cell Science • Supplementary information

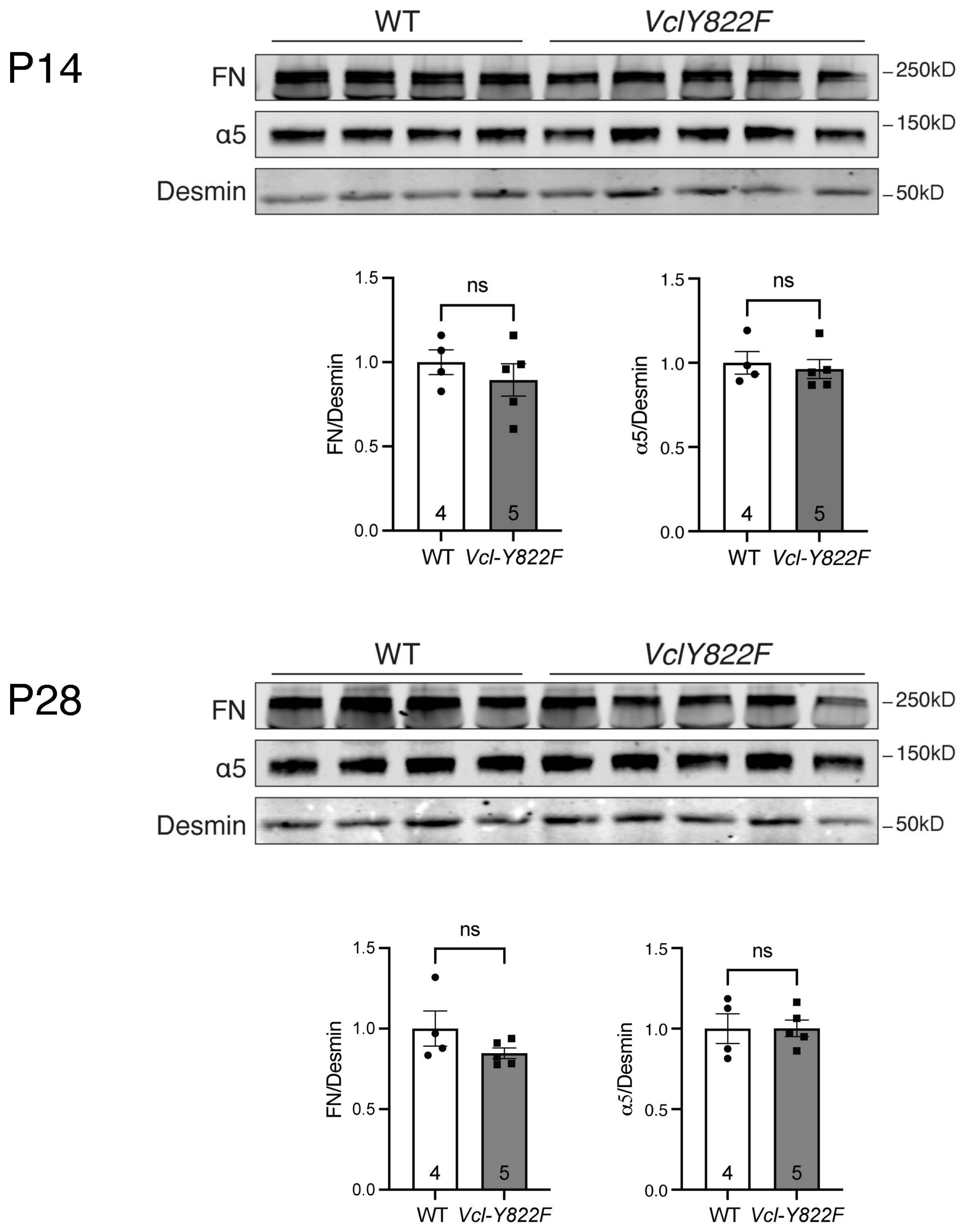

Fig. S2. No increase in FA or ECM protein expression in P14 and P28 *Vcl Y822F* mice. DOC insoluble hearts lysates from P14 (top) and P28 (bottom) WT and *Vcl Y822F* mice (n=4-5/genotype) were immunoblotted for Fibronectin (FN), α5 integrin (α5), and Desmin. Graphs show quantification of FN/Desmin and α5/Desmin ratios and comparison between WT and *Vcl Y822F* samples. Two-tailed, unpaired Student's t-test. Error bars represent SEM. ns, not significant.

Journal of Cell Science • Supplementary information

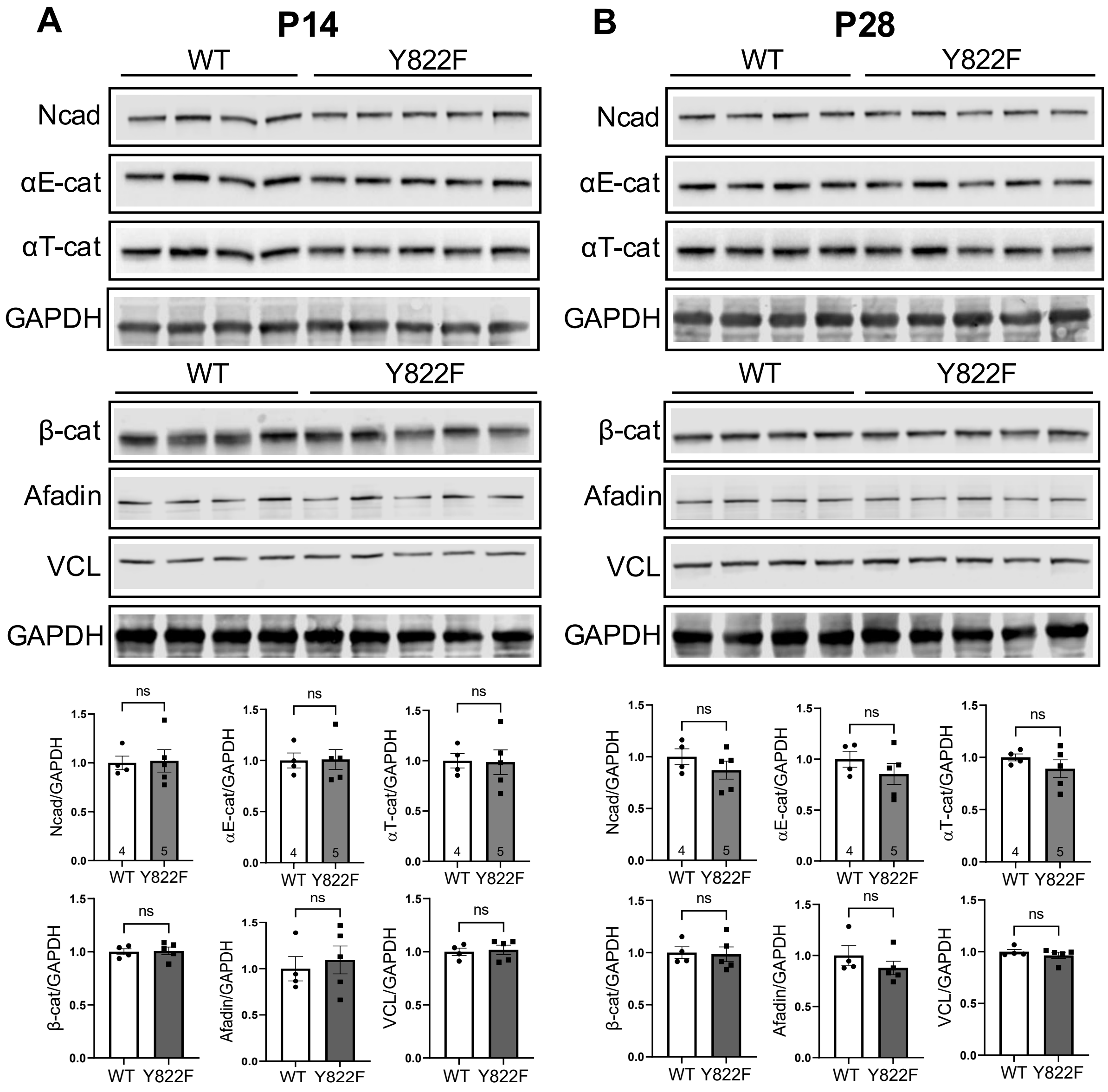

Fig. S3. N-cadherin/catenin complex protein levels do not change in *Vcl Y822F* hearts. (A, B) Western blots and quantitative analysis of heart lysates from WT (n=4) and *Vcl Y822F* (n=5) mice at P14 (A) and P28 (B). Blots probed for N-cadherin (Ncad), αE-catenin (αE-cat), αT-catenin (αT-cat), β-catenin (β-cat), Afadin, VCL, and GAPDH. Graphs show the ratio of N-cadherin/catenin complex proteins to GAPDH and comparison between WT and *Vcl Y822F* samples. Two-tailed, unpaired Student's t-test. Error bars represent SEM. ns, not significant.

Journal of Cell Science • Supplementary information

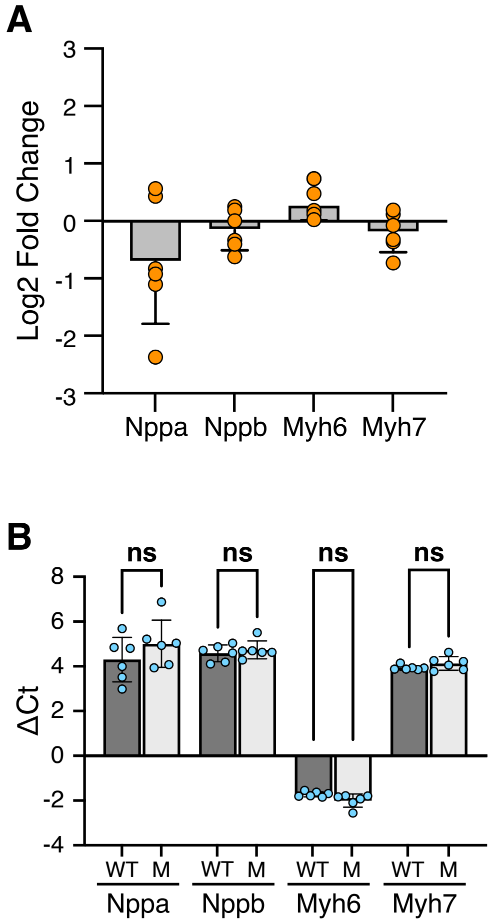

Fig. S4. Developmental gene expression is unchanged in Vcl Y822F hearts. (A) Gene expression measured by qRT-PCR from RNA isolated from 4-week-old WT and Vcl Y822F hearts (n=6 hearts/genotype). Orange circles mark the Log2(2-ΔΔCt) of individual Vcl Y822F hearts; columns and error bars define the mean and standard deviation. (B) Statistical significance between WT and Vcl Y822F qRT-PCR results in (A) was determined by comparing average ΔCt values. Blue circles mark the ΔCt values of each WT and Vcl Y822F mutant (M) sample; columns and error bars define the mean and standard deviation. Welch's t-test, ns = not significant, $p>0.05$.

Journal of Cell Science • Supplementary information

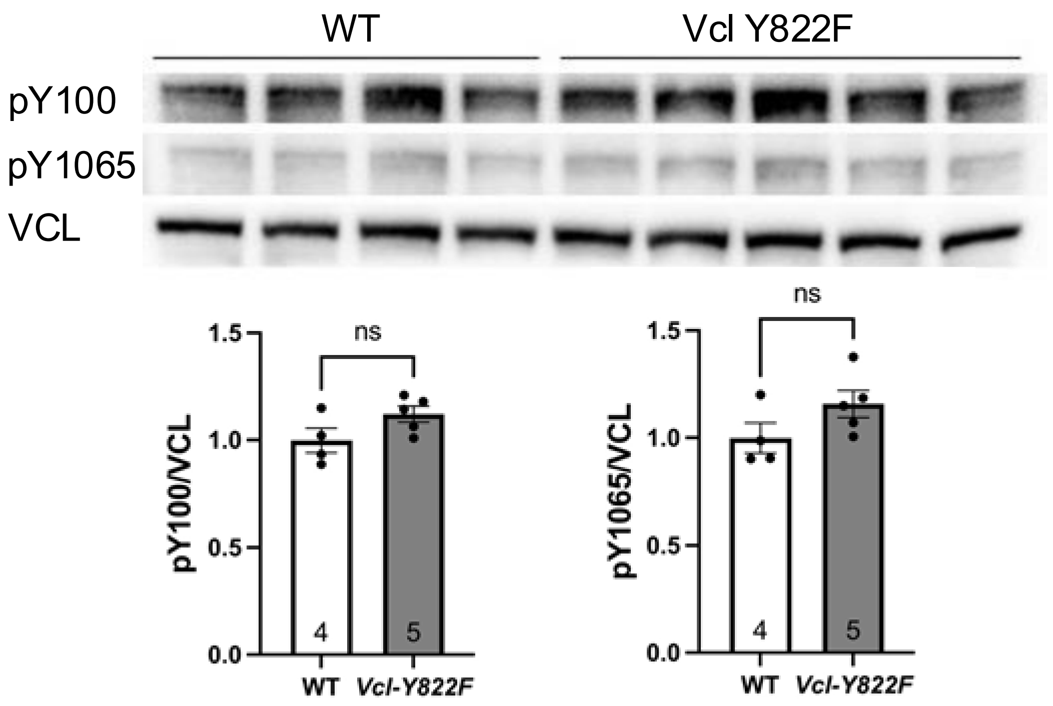

Fig. S5. Phosphorylation at VCL Y100 and Y1065 does not change in the *Vcl Y822F* hearts. Western blots and quantitative analysis of VCL pY100, VCL pY1065, and total VCL expression in P7 heart lysates from WT (n=4) and *Vcl Y822F* (n=5) mice. Graphs show the quantification of pY100/VCL and pY1065/VCL ratios and comparison between WT and *Vcl Y822F* samples. Two-tailed, unpaired Student's t-test. Error bars represent SEM. ns, not significant.

Journal of Cell Science • Supplementary information

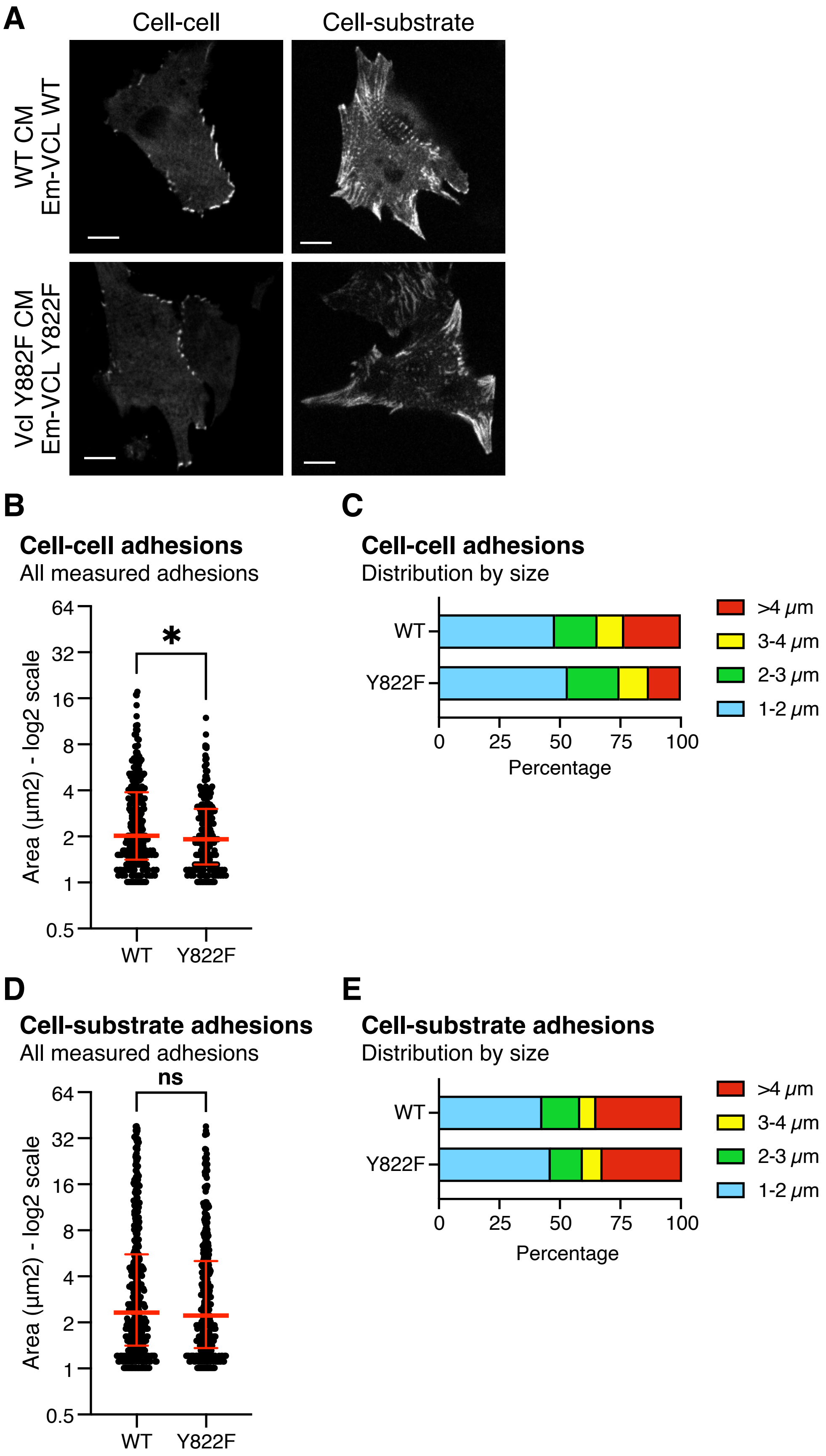

Fig. S6. Cell-cell adhesion area is reduced in Vcl Y822F CMs. (A, C) VCL adhesion size was measured along cell-cell (A) and cell-substrate (C) contacts in WT CMs expressing WT Emerald-VCL (WT) or Vcl Y822F CMs expressing Emerald-VCL Y822F (Y822F). Individual adhesions (WT: 273 cell-cell, 416 cell-substrate; Y822F: 221 cell-cell, 341 cell-substrate) were measured and plotted from three separate transfections from two independent cardiomyocyte preps. Adhesion area measurements were plotted on a log2 scale to visualize the data distribution. Median and interquartile range are marked with red lines. Mann-Whitney test, *p <0.05. (B, D) Adhesions from WT (B, data from A) and Y822F (D, data from C) were grouped by size, and each group was plotted as a percentage of the total.

Journal of Cell Science • Supplementary information

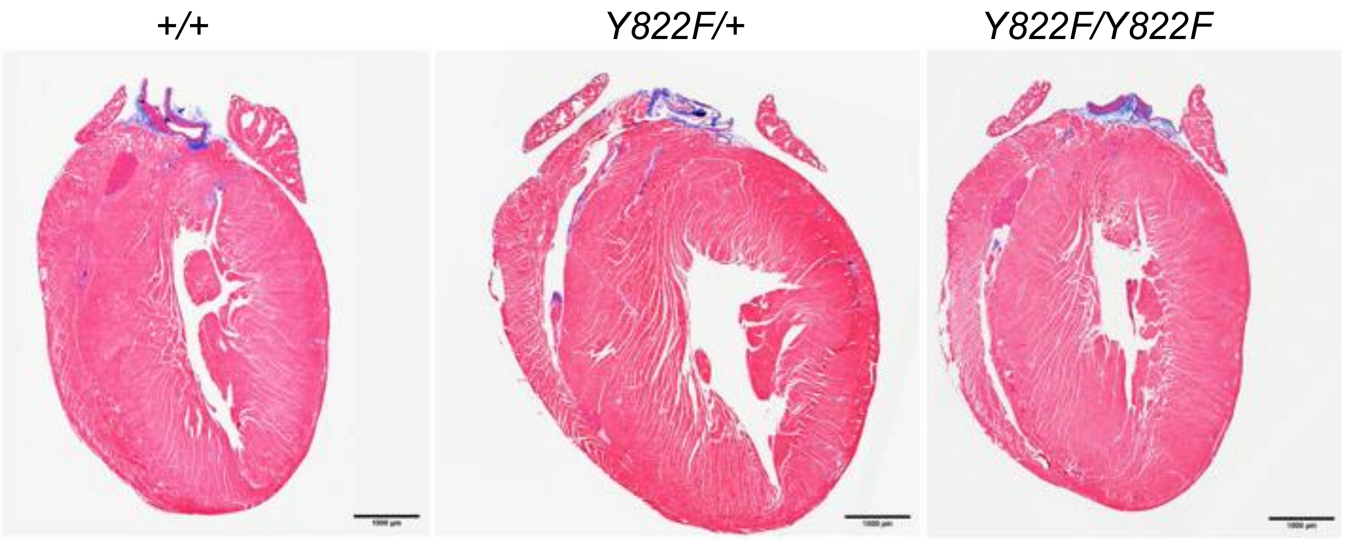

Supplemental Figure 7. No increase in fibrosis in *Vcl Y822F* hearts. (A) Representative Masson's Trichrome-stained heart sections from *Vcl +/+*, *Vcl Y822F/+,* and *Vcl Y822F/Y822F* female mice at 40 wks of age.

Journal of Cell Science • Supplementary information

Table S1. Echocardiographic indices of cardiac size and function

	WT	Y822 F/+	Y822 F/F
n	9	11	11
Gender(M/F)	4/5	7/4	4/7
Age(mo)	7	7	7
Heart rate(bpm)	471.44±3.95	472.27±4.05	470.82±2.75
LVAW;d(mm)	0.95±0.02	1.00±0.02	0.98±0.03
LVAW;s(mm)	1.10±0.03	1.15±0.02	1.14±0.02
LVID;d(mm)	3.80±0.11	3.96±0.10	3.75±0.07
LVID;s(mm)	2.58±0.11	2.82±0.09	2.68±0.08
LVPW;d(mm)	0.76±0.02	0.78±0.03	0.76±0.01
LVPW;s(mm)	1.14±0.02	1.15±0.02	1.10±0.03
CO (LV Trace) (mL/min)	18.96±0.82	18.60±1.18	14.68±0.72**
Diameter;d (LV Trace) (mm)	3.87±0.10	3.97±0.10	3.72±0.06
Diameter;s (LV Trace) (mm)	2.59±0.09	2.80±0.09	2.73±0.06
EF (LV Trace) (%)	62.30±1.27	56.91±1.65	52.80±1.79***
FS (LV Trace) (%)	33.12±0.86	29.51±1.09*	26.70±1.13***
LV Mass(mg)	119.38±5.81	134.13±6.13	119.61±3.78
LV Mass (Corrected) (mg)	95.50±4.65	107.30±4.90	95.69±3.03
LV Vol;d(μL)	62.79±4.42	69.12±4.17	60.48±2.70
LV Vol;s(μL)	24.77±2.61	30.58±2.37	26.88±2.00
SV (LV Trace) (μL)	40.29±1.88	39.28±2.31	31.19±1.54**
V;d (LV Trace) (μL)	65.17±3.92	69.32±4.03	59.17±2.20
V;s (LV Trace) (μL)	24.88±2.20	30.04±2.27	27.99±1.50

LVID, left ventricular internal dimension; LV vol, left ventricular volume; EF, ejection fraction; FS, fraction shortening; LVAW, left ventricular anterior wall; LVPW, left ventricular posterior wall; d, diastole; s, systole; CO, cardiac output; SV , stroke volume.
LV Trace: using Trace method. Values are represented as Mean±SEM.
One-way ANOVA with Dunnett's multiple comparison, *vs* WT group,
*$p < 0.05$, **$p < 0.01$, ***$p < 0.001$.

Journal of Cell Science • Supplementary information

Table S2. Vcl Y822F mouse model

Mouse CRISPR Editing

gRNA target sequence

Vcl **(PAM)**	5'-GGACTCAGGATATCGGATCC**TGG**-3'

Repair DNA oligonucleotide

Vcl Y822F, silent mutation ssDNA	5'-GGTGGAGGAGGCGGGAAGTCAGGCTCCTGAGGTTGGAAGGCTTCTCTGA CCTTGGCCACAGCTCC**gAG**GATCCG**AaA**TCCTGAGTCCAGGAAGCTCTTTT GCAGGCCTAAACACAAGACACTCATTTTCAGCTGGGTAGG-3'

Mouse genotyping

	Forward (5'>3')	Reverse (5'>3')	Product Size
Vcl Y822F (+EcoRV digest)	TACAGTCCAAAGGCATCCTCAC	GCACAAGTGCTGGCTTATGATT	514bp
SYR (Y-chromosome specific) used to determine sex of neonates	GCGCCCCATGAATGCATTTATG	CCCTCCGATGAGGCTG	221bp

Table S3. Guide RNA sequence and its top off-targeting sites

Guide RNA sequence (5'-3')	PAM	Off-target	Efficiency Score
GGACTCAGGATATCGGATCC	TGG	91.2	47.2

Sequence	PAM	Score	Gene	Locus
GGACTCAGGATATCGGATCC	TGG	100.0	Vcl (ENSMUSG00000021823)	chr14:+21022039
GGACTCATGATATAGGATCC	CGG	1.0		chr1:+129999031
GACCTCAGGGTACCGGATCC	TGG	0.6		chr18:+77559597
GTCCTCAGGGTATCGGATTC	CGG	0.6		chr8:+13180080
GGAGACAGGATATTGGATCC	CAG	0.5		chr4:-55689662
GGATGCAGGGTATCTGATCC	TAG	0.4		chr8:+64646519
GGACTCATGACATCGTATCC	TGG	0.3		chr15:-59557613
TGACACAAGATATGGGATCC	TAG	0.3		chr15:-71436166
GAACTCAGACTATCCGATCC	CAG	0.3		chr6:-51753296
AGAGGCAGGATATTGGATCC	TAG	0.3		chr7:+140066581
GTGCTCAGGATCTCTGATCC	AGG	0.2		chr10:-86424400
